# Metagenomic Approach to Characterizing Disease Epidemiology in a Disease-Endemic Environment in Northern Thailand

**DOI:** 10.3389/fmicb.2019.00319

**Published:** 2019-02-26

**Authors:** Ratree Takhampunya, Achareeya Korkusol, Chalermpol Pongpichit, Komsan Yodin, Artharee Rungrojn, Nitima Chanarat, Sommai Promsathaporn, Taweesak Monkanna, Sasikanya Thaloengsok, Bousaraporn Tippayachai, Naruemon Kumfao, Allen L. Richards, Silas A. Davidson

**Affiliations:** ^1^Department of Entomology, US Army Medical Directorate of the Armed Forces Research Institute of Medical Sciences (USAMD-AFRIMS), Bangkok, Thailand; ^2^Bo Kluea Hospital, Nan, Thailand; ^3^Viral and Rickettsial Diseases Department, Naval Medical Research Center, Silver Spring, MD, United States

**Keywords:** metagenomic, bacterial community, disease epidemiology, disease transmission, scrub typhus, undifferentiated febrile illness

## Abstract

In this study, we used a metagenomic approach to analyze bacterial communities from diverse populations (humans, animals, and vectors) to investigate the role of these microorganisms as causative agents of disease in human and animal populations. Wild rodents and ectoparasites were collected from 2014 to 2018 in Nan province, Thailand where scrub typhus is highly endemic. Samples from undifferentiated febrile illness (UFI) patients were obtained from a local hospital. A total of 200 UFI patient samples were obtained and 309 rodents and 420 pools of ectoparasites were collected from rodents (*n* = 285) and domestic animals (*n* = 135). The bacterial 16S rRNA gene was amplified and sequenced with the Illumina. Real-time PCR and Sanger sequencing were used to confirm the next-generation sequencing (NGS) results and to characterize pathogen species. Several pathogens were detected by NGS in all populations studied and the most common pathogens identified included *Bartonella* spp., *Rickettsia* spp., *Leptospira* spp., and *Orientia tsutsugamushi*. Interestingly, *Anaplasma* spp. was detected in patient, rodent and tick populations, although they were not previously known to cause human disease from this region. *Candidatus* Neoehrlichia, *Neorickettsia* spp., *Borrelia* spp., and *Ehrlichia* spp. were detected in rodents and their associated ectoparasites. The same *O. tsutsugamushi* genotypes were shared among UFI patients, rodents, and chiggers in a single district indicating that the chiggers found on rodents were also likely responsible for transmitting to people. Serological testing using immunofluorescence assays in UFI samples showed high prevalence (IgM/IgG) of *Rickettsia* and *Orientia* pathogens, most notably among samples collected during September–November. Additionally, a higher number of seropositive samples belonged to patients in the working age population (20–60 years old). The results presented in this study demonstrate that the increased risk of human infection or exposure to chiggers and their associated pathogen (*O. tsutsugamushi*) resulted in part from two important factors; working age group and seasons for rice cultivation and harvesting. Evidence of pathogen exposure was shown to occur as there was seropositivity (IgG) in UFI patients for bartonellosis as well as for anaplasmosis. Using a metagenomic approach, this study demonstrated the circulation and transmission of several pathogens in the environment, some of which are known causative agents of illness in human populations.

## Introduction

Most public health surveillance systems and laboratories rely on serological and molecular assays that were developed to detect specific pathogens. However, conventional laboratory assays are often ineffective at detecting all causative agents of disease. Studies have shown that 40% of gastroenteritis cases (Finkbeiner et al., 2008) and as many as 60% of encephalitis cases (Ambrose et al., 2011) went undetected by conventional laboratory testing. Pathogens can go undetected if they are novel or are not known to previously occur in an area. There are many examples of the emergence of novel pathogens or reemergence of known organisms in new places where the available surveillance systems were inadequate, such as occurred with outbreaks of H7N9 influenza (Gao et al., 2013), Middle East respiratory syndrome coronavirus (MERS-CoV) (van Boheemen et al., 2012; Kindler et al., 2013), and the severe acute respiratory syndrome (SARS) outbreak in 2003 (Wang and Jolly, 2004).

Conventional diagnostic tests used by most reference laboratories require culture, microscopy, serology, and polymerase chain reaction (PCR). Such tools are useful for pathogen detection but only if culture conditions, test sensitivity, and primers are compatible and suitable for the microbial target. Other molecular approaches can be used to capture a wider range of pathogenic species such as multiplex PCR that targets highly conserved DNA regions or multiplex assays that target many of the most common pathogens known to cause similar symptoms. However, it is worth noting that even when multiplex assays are used, pathogens not included in the multiplexing may go undetected. The use of 16S rDNA was first proposed by Woese and Fox (1977) and Woese et al. (1990) as a tool for the molecular identification and characterization of microorganisms. The 16S rDNA gene is highly conserved among prokaryotes and some parts of its sequence are hypervariable between species, which makes it an ideal marker for species identification and for understanding evolutionary relationships (Gill et al., 2006; Sogin et al., 2006; Dethlefsen et al., 2008; McInerney et al., 2008; Tringe and Hugenholtz, 2008; Sunagawa et al., 2009). Metagenomics allows for comparisons of genetic material from multiple samples. One of the most common metagenomic approaches is deep amplicon sequencing (DAS), which employs universal primer to amplify parts of the 16S rRNA gene from specimens. A major benefit of metagenomics is the simultaneous detection of all microorganisms in clinical samples without prior knowledge of their identities. In addition, metagenomics has the potential to detect rare and novel pathogens. Current surveillance assays are limited in their ability to detect the emergence of novel pathogens or ones not previously known to be present in a given region. Metagenomic approaches can fulfill such gaps by identifying unknown etiological agents and assisting in the development of a new test for pathogen detection (Miller et al., 2013; Mokili et al., 2013; Wan et al., 2013).

Metagenomic approaches are especially suitable for zoonotic diseases. It is estimated that more than 60% of human pathogens are of animal origin (Taylor et al., 2001). Rodents are major reservoirs that account for a wide range of emerging zoonotic diseases in humans and livestock (Jones et al., 2008; Meerburg et al., 2009). Co-infection of multiple pathogens within individual rodents is frequently observed and the interaction between pathogens can have significant effects (Cox, 2001). Such co-infections can cause rodents to be more or less susceptible to other microparasites (Tadin et al., 2012). Generally, multiple infections in wildlife can increase disease severity in a host (Lello et al., 2005), affecting the survival and reproduction of animal hosts (Davidar and Morton, 2006; Holmstad et al., 2008). Disease surveillance in rodents and other wildlife can provide important information for public health preparedness. Surveillance can also be used to measure biodiversity and disease emergence which are both directly linked to the stability of ecosystems (Keesing et al., 2010; Grogan et al., 2014). Metagenomic approaches combined with NGS can be powerful tools to disentangle complex patterns of pathogen transmission among ectoparasites, animal reservoirs, and humans. For example, NGS has been used to perform blood meal analysis to determine the wide-range of animals that vectors feed on and possible reservoirs (Alcaide et al., 2009). NGS has also been useful in finding unexpected pathogens not normally associated with particular vectors (Vayssier-Taussat et al., 2013) and has been used to show the genetic diversity of bacteria that are specific to certain animal hosts and vectors (Pierlé et al., 2014; Swei et al., 2015). Such information can be used to correlate infections in people with important vectors and reservoir hosts.

In this study, metagenomics and NGS technology were used to characterize human (patients with undifferentiated febrile illness (UFI)), reservoir host (rodents and small mammals), and ectoparasite (chiggers, ticks, fleas, and lice) populations for bacterial pathogens. All samples were collected from Nan province in northern Thailand. Since all samples were from the same sites, bacteria could be compared from different populations to determine potential vectors and reservoirs. Nan province is highly endemic for scrub typhus, caused by the agent *Orientia tsutsugamushi*, and one of the major goals of this study was to determine the etiology and transmission dynamics of scrub typhus in the area. Another goal was to identify other bacterial pathogens that were under-reported or not previously known from this region. NGS results were verified by conventional methods such as real-time PCR, PCR, and DNA sequencing to confirm the pathogenic potential of detected bacteria and to better characterize those important pathogens to the species level. In addition, serological tests were performed to determine the seroprevalence and the history of human exposure to the pathogens detected by the NGS approach. The in-depth characterization of bacteria performed in this study from humans, animal hosts, and ectoparasites allowed us to determine the transmission dynamics of pathogens and identify several new and previously unreported pathogens from this area.

## Materials and Methods

### Ethics Statement

Rodents were trapped according to the institutional animal collection protocol entitled “Field Sampling of Small Mammal (Orders: Erinaceomorpha, Soricomorpha, Scandentia, Macroscelidea, and Rodentia) Populations to Support Zoonotic Disease Surveillance and Ectoparasite Collection” (PN# 12–06), reviewed and approved by the USAMC-AFRIMS Institutional Animal Care and Use Committee (IACUC). All sampling procedures and experimental manipulations were reviewed and approved as part of the animal collection protocol (PN# 12–06). Research was conducted in compliance with the Animal Welfare Act and other federal statutes and regulations related to animals and experiments involving animals, and adhered to principles outlined in the “Guide for the Care and Use of Laboratory Animals,” NRC Publication, 2011 edition.

### Undifferentiated Febrile Illness (UFI) Patients

A total of 200 individual UFI patients’ coded specimens were received from Bo Kluea hospital, Nan province, Thailand. Samples were from outpatients and inpatients presenting to Bo Kluea hospital with UFI and suspicion of scrub typhus infection during February–November 2017. Residual whole blood and serum samples from routine laboratory testing were coded and sent to the Department of Entomology to be tested for the possible causative agent of UFI as well as for scrub typhus or murine typhus infection, caused by *Rickettsia typhi*. The protocol was determined on February 01, 2016 by WRAIR Human Subjects Protection Branch (HSPB) to be research not involving human subjects for this investigation, since the work described herein involves the use of existing, coded specimens wherein investigators will not receive associated identifiable data, the project did not require a review by the Institutional Review Board (IRB) and 45 CFR 46 and 32 CFR 219 does not apply.

### Study Locations

**FIGURE 1:**
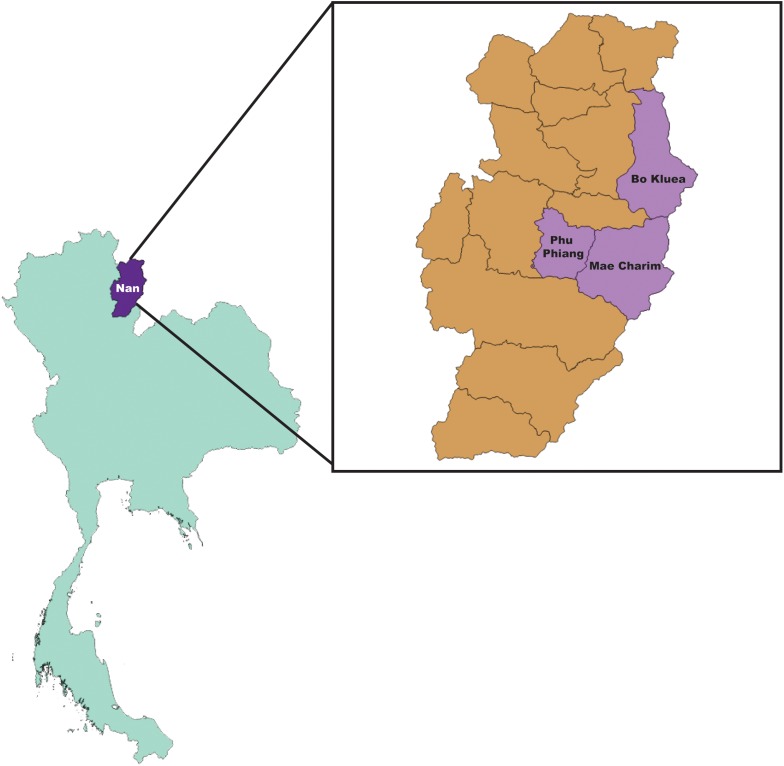
Map of collection sites; Bo Kluea, Mae Charim, and Phu Phiang Districts, Nan province, Thailand.

Rodents and ectoparasites were collected during the wet (June–September) and dry (November–April) seasons in Bo Kluea, Mae Charim, and Phu Phiang districts of Nan province, Thailand in 2014–2018 (Figure 1 and Table 1). Ectoparasites were also collected from domesticated mammals (dogs, cats, and cattle). All study sites were on private land, and permission was obtained from each of the owners to conduct research on their land. None of the field studies involved endangered or protected species. Rodents were captured using live traps baited with bananas, palm fruit, or dried fish, and were collected from orchards, palm and rubber plantations, cultivated rice-fields, grassland areas, edges of dense forest, stream margins, and around dwellings. Traps were set for 3–5 nights and were checked early in the morning. Captured rodents were removed from the traps, euthanized using carbon dioxide, and processed immediately at the site of collection. Blood, serum, and tissue samples (liver, spleen, kidney, and lung) were collected and stored on dry ice. Ears were removed and stored in 70% ethanol for chigger collection. All tissues were then transported to the AFRIMS laboratory for further processing. All rodents were later identified to the species level as described previously (Muul, 1979).

**Table 1:** Summary of sample description, sample size, pooling, and NGS coverage.

Sample types	Host	Number of samples studied	Number of NGS pool	Number of sample(s) per NGS pool	Collection sites in Nan province, Thailand	Year of collection	Number of reads in OTUs (minimum–maximum)	Mean number of read ± SD
UFI patients	N/A	200	23	4–14	Bo Kluea Hospital	2017	17,879–123,232	69,493.39 ± 37,001.57
Rodents	N/A	309	64	1–13	Bo Kluea, Mae Charim, Phu Phiang	2014, 2017, 2018	1,583–133,878	67,858.53 ± 32,222.56
Chiggers	Rodents	199	43	1–12	Bo Kluea, Mae Charim, Phu Phiang	2014, 2017, 2018	2,810–56,552	30,355.47 ± 15,008.78
Ticks	Rodents	59	17	1–11	Bo Kluea, Mae Charim, Phu Phiang	2014, 2017, 2018	30,968–130,783	56,570.71 ± 21,921.34
Fleas	Rodents	23	8	1–8	Bo Kluea, Mae Charim	2014, 2018	24,890–131,129	75,680.25 ± 35,071.38
Lice	Rodents	4	4	1	Bo Kluea, Mae Charim, Phu Phiang	2014, 2017, 2018	55,352–93,336	74,310.25 ± 16102.85
Ticks	Domesticated mammals	35	8	1–13	Mae Charim	2014	30,026–70,664	45,411.88 ± 12,203.16
Fleas	Domesticated mammals	88	12	1–11	Mae Charim	2014	19,163–106,928	49,278.42 ± 25,610.98
Lice	Domesticated mammals	12	4	2–4	Mae Charim	2014	6,773–160,184	59,614.00 ± 68,479.14

*N/A, not available.*

### Genomic DNA Extraction

#### UFI Whole Blood

Genomic DNA was extracted from whole blood samples by automated extraction machine, a QIAsymphony^®^ SP instrument (Qiagen, Hombrechtikon, Switzerland) with QIAsymphony^®^ DNA Mini Kit (Qiagen, Germany). For each patient, 250 μl of whole blood was used for the DNA extraction with DNA Blood 200 DSP protocol. DNA was eluted in 50 μl and stored at −20°C until use. Ultrapure DNA/RNA-free distilled water was also included in every extraction procedure as an extraction control.

#### Rodent Tissue

Spleen and kidney tissues from each rodent were cut into pieces (∼3 mm in diameter) and added to 230 μl of ATL Tissue Lysis Buffer and 20 μl of Proteinase K solution (20 mg/ml), then incubated at 55°C for 1 h or until the tissues were homogenized. A total volume of 250 μl homogenized solution was then used for DNA extraction on the QIAsymphony^®^ SP with QIAsymphony^®^ DNA Mini Kit and Tissue HC 200 DSP protocol. The DNA was eluted in 200 μl and stored at −20°C until use. Ultrapure DNA/RNA-free distilled water was also included as an extraction control.

### Ectoparasite Morphological Identification and DNA Extraction

Ectoparasites (chiggers, ticks, fleas, and lice) collected from rodents and small mammals were morphologically identified and pooled by genus, the host species they were collected from, and ectoparasitic stage. Chiggers were identified to genus level using a taxonomic key (Nadchatram, 1974). Other ectoparasites (fleas, ticks, and lice) were identified morphologically (Hopkins and Miriam, 1953; Tanskull and Inlao, 1989; Durden and Musser, 1994) and pooled by the host species they were collected from, type, stage, and gender of ectoparasites. Each pool was subjected to genomic DNA extraction using a modified protocol of QIAamp DNA Mini Kit (Qiagen). Briefly, ectoparasites in 180 μl of ATL buffer were punctured with a fine needle under a stereomicroscope to release the tissue from the hard chitin exoskeleton prior to adding 20 μl of Proteinase K solution (20 mg/ml). Samples were then incubated at 55°C for 1 h or until the ectoparasites were homogenized. A volume of 200 μl of AL buffer was added to the sample and the sample mixed and incubated at 70°C for 10 min. Then 100 μl of absolute ethanol was added to precipitate DNA. The solution was transferred to a QIAamp DNA column then centrifuged at 8,000 rpm for 1 min. The supernatant was discarded. DNA was washed twice with 500 μl of AW1 and AW2, respectively. The DNA was eluted at 50 μl of AE buffer and stored at −20°C until used. Ultrapure DNA/RNA-free distilled water was also included as an extraction control.

### Amplification of Bacterial 16S DNA

Following DNA extraction of patient whole blood and rodent tissue, bacterial-specific 16S rDNA (V3–V4, a 550 bp fragment) was amplified in three replicates using the universal bacterial primer set; 16S amplicon PCR Forward primer (TCGTCGGCA GCGTCAGATGTGTATAAGAGACAG CCTACGGGNGGCW GCAG) and Reverse primer (GTCTCGTGGGCTCGGAG ATGTGTATAAGAGACAG GACTACHVGGGTATCTAATCC) (gene- specific sequences are underlined). PCRs were performed in a 20-μl volume containing 5 μl (1–100 ng/μL) of DNA template, 400 nM each primer, 200 μM dNTPs, 1.5 mM MgCl_2_, 1× PCR buffer, and 0.4 U of iProof High-Fidelity DNA Polymerase (Bio-Rad, Hercules, CA, United States). Amplification was performed using a T100 DNA thermal cycler (Bio-Rad) under the following conditions: initial denaturation at 98°C for 3 min; 40 cycles of 98°C for 10 s, 60°C for 20 s, and 72°C for 30 s; and a final extension at 72°C for 5 min.

For DNA from all ectoparasites, a fragment of 16S rDNA (V1–V6) region (1,016 bp) was amplified in triplicate in a first-round PCR using primers; 27F-Y (5′-GAGTTTGATCCTGGCTYAG-3′), 1061R (5′-CRRCACGAGCTGACGAC-3′) (Ong et al., 2013), and 2.5 μM MidBlocker oligonucleotide to inhibit 16S *Candidatus* Midichloria mitochondrii amplification (Gofton et al., 2015b). The reaction was performed in a 20-μl volume containing 3 μl of ectoparasite DNA, 400 nM each primer, 200 μM dNTPs, 1.5 mM MgCl_2_, 1× PCR buffer, and 0.4 U of iProof High-Fidelity DNA Polymerase (Bio-Rad). Amplification was performed using a T100 DNA thermal cycler (Bio-Rad) under the following conditions: initial denaturation at 98°C for 3 min; 40 cycles of 98°C for 10 s, 60°C for 20 s, and 72°C for 30 s; and a final extension at 72°C for 5 min. The second amplification was performed as described above for human and rodent samples. Negative control PCR reactions were included in every experimental run using Ultrapure DNA/RNA-free distilled water in place of DNA template. PCR reactions were also performed with eluates from mock DNA extractions.

Three PCR products from each sample were pooled and cleaned using AMPure magnetic bead-based purification system (Beckman Coulter, United Kingdom) following the manufacturer’s instructions. Purified PCR products were eluted and quantified using the Quant-iT PicoGreen dsDNA assay (Invitrogen Life Technologies, MA) according to the manufacturer’s protocol. Each purified PCR was normalized and then pooled again with other purified PCR products from other samples by; (i) gender and age group for UFI patients, (ii) season of collection, location (sub-district/district), and rodent genera/species for rodents and rodent chiggers, and (iii) the host type they were collected from, genus/species and stages of ectoparasites for all other ectoparasites (ticks, fleas, and lice) collected from rodents and domesticated mammals (dogs and cows). Additional details on sample pooling for NGS are provided in Supplementary Table S2.

### Library Preparation and High Throughput Sequencing

#### Indexing Samples

The dual indices and Illumina sequencing adapters were attached to pooled, purified PCR products using the Nextera XT Index Kit following the manufacturer’s protocol (Illumina). Index control reaction: combination of index primers that were not used with samples, was also included with PCR grade water as template. The number of reads recovered from these particular index combinations should be used to filter the cross-contaminations between indexed PCR primers and to identify errors in an Illumina sample sheet.

#### Library Clean Up, Normalization and Pooling

The final products were cleaned using Agencourt AMPure XP beads. The purity of the libraries was checked on the QIAxcel Advanced System (Qiagen) with a QIAxcel DNA High Resolution Cartridge. Purified amplicon libraries were quantified using the Qubit dsDNA HS Assay Kit (Invitrogen). DNA concentration was calculated and normalized to reach 4.0 nM for each library. Five microliters of DNA from each library were pooled (each NGS pool had 29–78 DNA libraries) for a NGS run (1–5 runs in total). Pooled libraries were denatured and diluted to a final concentration of 8 pM with a 10% PhiX (Illumina) control. Sequencing was performed using the MiSeq Reagent Kit V3 on the Illumina MiSeq System.

### Data Analysis

The sequence reads generated by the 16S rRNA on MiSeq sequencers were processed on the CLC Genomics workbench v 11.0.1 (Qiagen, Aarhus A/S^1^). High-throughput sequences were imported into CLC Genomics Workbench according to quality scores of Illumina pipeline 1.8. In order to achieve the highest quality sequences for clustering, paired reads were merged in CLC microbial genomics module v3.0 using default settings (mismatch cost = 1; minimum score = 40; gap cost = 4 and maximum unaligned end mismatch = 5). Primer sequences were trimmed from merged reads using parameters (trim using quality scores = 0.01, trim ambiguous nucleotides = 2, and discard read length shorter than 150 bp). Samples were removed from analysis if the number of reads was less than 100 or less than 25% from the median (the median number of reads across all samples).of minimum read from the median. Chimeric sequences were detected and removed. Filtered sequences were clustered into operational taxonomic units (OTUs) according to a threshold of 97% sequence identity. All such processes were performed using CLC microbial genomics module v3.0. Reference OTU data used in the present study were downloaded from the Greengenes database V13.8 (DeSantis et al., 2006) and SILVA 16S V132 (Quast et al., 2013). Alpha rarefaction curve plots were generated among samples using CLC Microbial Genomics Module v3.0 with default parameter settings (minimum depth = 1, maximum depth = 100,000 and number of point = 20).

### Pathogen Characterization by PCR Amplification and Sanger Sequencing

Real-time PCR and PCR assays were performed on positive NGS pools to confirm the detection of pathogen and the taxonomic species assignment generated by NGS analysis. The detail of assays and target gene(s) for selected pathogens was provided as online Supplementary Data (Supplementary Table S1) (Norman et al., 1995; Barbour et al., 1996; Horinouchi et al., 1996; Schwaiger et al., 2001; Scoles et al., 2001; Smythe et al., 2002; Park et al., 2004; Klee et al., 2006; Chmielewski et al., 2009; Colborn et al., 2010; Ganoza et al., 2010; Billeter et al., 2011; Parola et al., 2011; Diaz et al., 2012; Lalzar et al., 2012; Shakya et al., 2013; Gofton et al., 2015a; Pereira et al., 2018). For all real-time PCR, the reaction consisted of 1X Platinum quantitative PCR SuperMix-UDG (Invitrogen) using standard real-time PCR conditions with primer/probe concentrations and annealing temperatures as indicated in Supplementary Table S1. For conventional PCR, the assay was carried out in a 50 μl reaction volume containing 0.5 U of iProof High-Fidelity DNA Polymerase, 200 μM dNTPs, MgCl_2_ and primer concentration as indicated (Supplementary Table S1). The PCR conditions consisted of 98°C for 3 min, followed by 40 cycles of 98°C for 10 s, annealing temperature for 30 s, and 72°C for 45 s.

### Estimating the Prevalence of Each Pathogen in Samples Studied

After performing NGS analysis of pooled samples, all samples in each NGS-positive pool for potential pathogenic bacteria were individually tested by their respective confirmatory assays using either real-time PCR or conventional PCR as indicated in Supplementary Table S1. Any positive signal was then confirmed by DNA sequencing by the Sanger method for species characterization. The prevalence rate for each pathogen was calculated based on the number of positive samples verified by confirmatory assays in the total number of samples studied. For some pathogens including *O. tsutsugamushi* and *Bartonella* spp., all samples were screened as routine tests. Therefore, the prevalence rate was calculated based on the number of combined positive samples detected by the NGS analysis then confirmatory assays and routine screening tests.

### DNA Sequence and Phylogenetic Analysis

PCR amplicons were purified using the QIAquick^®^ PCR Purification Kit (Qiagen) according to the manufacturer’s instructions. The PCR products were cycle-sequenced using an ABI BigDye^TM^ Terminator v3.1 Cycle Sequencing Kit, ethanol precipitated, and run on a SeqStudio Genetic Analyzer (Applied Biosystems Thermo Fisher, Thailand). Sequences of each sample and pathogen were assembled using Sequencher^TM^ ver. 5.1 (Gene Codes Corp., Ann Arbor, MI, United States). The pathogen sequences were aligned with reference sequences retrieved from the GenBank database using the MUSCLE codon alignment algorithm (Edgar, 2004). A maximum likelihood phylogenetic tree was then constructed from bacterial target gene(s) (Supplementary Table S1) using the best fit model of nucleotide substitution with bootstrapping (1000 replicates) in MEGA 6 (Tamura et al., 2013).

### Serological Tests

#### Immunofluorescence Assay (IFA)

Scrub typhus and typhus group and spotted fever group of rickettsial diseases IFA tests were used to detect group-specific IgM antibodies against scrub typhus orientiae, and murine typhus and the spotted fever group of rickettsiae. The *Rickettsia* Screen IFA IgM Antibody Kit was used following the manufacturers’ instruction (Fuller Laboratories, Fullerton, CA, United States). The assay is intended for the simultaneous detection and semi-quantitation of IgM human antibody to both typhus group (TG) and spotted fever group (SFG) rickettsiae. An *O. tsutsugamushi* IFA IgM Antibody Kit (Fuller) included 4 strains (Boryong, Gilliam, Karp, and Kato) in one well. Positive reaction appears as bright staining (at least 1+) of positive control cut-off level in any of the four antigens areas. *Bartonella henselae* and *Anaplasma phagocytophilum* IFA tests were conducted for the detection of human IgG antibodies from serum using commercial kits [*B. henselae* IFA Human IgG Antibody Kit, *A. phagocytophilum* (HGA) IFA IgG Antibody Kit, Fuller]. Serum screening dilutions for *B. henselae* and *A. phagocytophilum* were 1:64 and 1:80, respectively.

In-house *B. quintana* IFA assay was prepared by CDC, Fort Collins (CO, United States) for testing the presence of human IgG against *B. quintana*. The protocol has been published previously (Iralu et al., 2006; Myint et al., 2011). Briefly, the human serum (1:32 dilution) was added to a slide fixed with *B. quintana* antigen, prepared by infecting Vero E6 cells with the bacteria. The slide was then incubated in a moist chamber at 35°C for 30 min, washed with PBS for 10 min, rinsed with distilled water, and air dried. Anti-human fluorescein isothiocyanate-labeled IgG conjugate was added to the slide which was processed as before. The slide was then mounted and read on a fluorescent microscope. All positive samples were then serially diluted in PBS, and an IFA-endpoint titer was determined using the same procedure; the end cut-off value for *B. quintana* was a titer greater than 1:200.

#### Enzyme-Linked Immunosorbent Assay (ELISA)

The determination of serological reactivity to *O. tsutsugamushi* 56-kDa recombinant protein was performed by in-house ELISA as previously described (Jiang et al., 2004). The ELISA plates were coated with 4 recombinants of *O. tsutsugamushi* 56-kDa protein from Karp, Gilliam, Kato, and TA763 genotypes. Patient sera were diluted at 1:100 with PBS for screening procedure. Samples considered positive (>0.5 OD) were further titered to determine their endpoint. The titer procedure was performed by diluting the positive sera by a factor of 4 (1:100, 1:400, 1:1,600, and 1:6,400) and tested again with the same procedure. If the sample had a total absorbance for all 4 dilutions of 1.00 or greater for the net OD, then the sample was considered reactive and the titer value was the inverse of the highest dilution with the OD of 0.2 or greater.

Enzyme-linked immunosorbent assay for the detection of IgG class antibody against *R. typhi* and spotted fever group *Rickettsia* in human serum was performed using commercial kits (*R. typhi* EIA IgG Antibody Kit, Spotted Fever *Rickettsia* IgG EIA Antibody Kit, Fuller). The kits utilized a group-specific lipopolysaccharide (rLPS) antigen extracted from spotted fever group *Rickettsia* species and a species-specific protein (r*Omp*B) purified from *R. typhi*.

### Statistical Analysis and Data Visualization

All statistical analyses (linear regression and two-way ANOVA tests) were performed using GraphPad Prism version 5.04 for Windows (GraphPad Software, San Diego, CA, United States ^2^). Some graphical illustrations presented in this study were performed in the R environment for statistical computing (Wickham, 2009). A nucleotide distance matrix was generated using “DNADist DNA Distance Matrix” in BioEdit (Hall, 1999). Maps used in this study were created by QGIS software (QGIS Development Team, 2009. QGIS Geographic Information System. Open Source Geospatial Foundation^3^).

## Results

### Sample Collection and NGS Procedure

**FIGURE 2:**
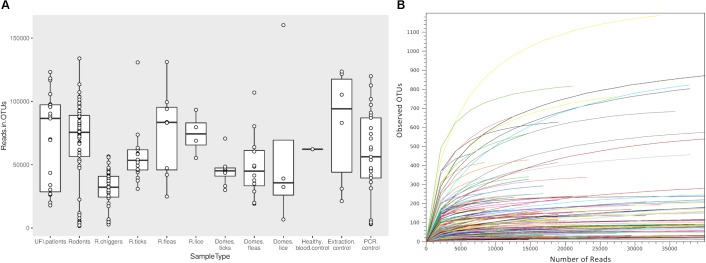
Box plot showing the distribution of number of reads from each NGS pool used for OTU assignment in this study **(A)**. Rarefaction curves for all samples included in this study **(B)**. The curves show the number of taxonomic units (OTUs) as a function of the number of sequences, indicating the sampling completeness. R, rodents; Domes, domesticated mammals.

Samples included in this study were from UFI patients (*n* = 200), rodents and small mammals (*n* = 309), rodent-associated ectoparasites (chiggers = 199 pools, ticks = 59 pools, fleas = 23 pools, and lice = 4 pools), and ectoparasites collected from larger animals including dogs, cats, and cattle (ticks = 35 pools, fleas = 88 pools and lice = 12 pools) (Table 1). Samples were collected mainly from Bo Kluea and from nearby districts of Nan province (Figure 1), Thailand. UFI samples were from inpatients and outpatients visiting the hospital with symptoms similar to scrub typhus infection or fever of unknown origin throughout the year 2017. The sampling of rodents and ectoparasites took place twice each year (wet and dry seasons) in 2014 and 2017, and only once in 2018 (dry season). Each sample was amplified in triplicate reactions to minimize PCR bias (Acinas et al., 2005; Sipos et al., 2007; Aird et al., 2011) and PCR products from the three reactions were pooled for each sample and purified before pooling with other samples for library preparation before NGS. All totaled, 929 samples were pooled according to their sample type, area of collection, season of collection, and host species into 183 NGS pools (Table 2). After NGS quality control procedures, 13,225,584 16S sequences from the field-collected samples and 38 control samples (6 extraction controls, 25 PCR controls, and 7 index controls) were used for analysis. From the 38 control samples, 153 bacterial genera were detected and then subtracted from the field samples’ 16S sequence dataset before conducting downstream analysis. These bacterial genera were considered to be contaminants from molecular reagents, the environment (water), or from cross-contamination during sample processing and between NGS runs (Salter et al., 2014). The number of pass-filtered raw reads per NGS pool ranged from 24,939 to 627,297, with the highest read (1,625,690) belonging to an ectoparasite pool collected from domesticated mammals (mean ±*SD* = 294,518 ± 152,314). The number of reads per NGS pool used in OTU assignment ranged from 1,583 to 160,184 (mean ±*SD* = 110,802 ± 34,095) (Table 1 and Figure 2A). The majority of samples with low numbers of reads were from chigger samples. Overall, 7.8% of OTUs (1,032,389 reads) were unclassifiable at the phylum level. Rarefaction curves demonstrated that sequence data for all samples approached completeness as indicated by the curve plateaus (Figure 2B). These data suggest that most bacterial profiles of all samples studied were nearly complete.

**Table 2:** Eleven pathogenic bacterial genera detected in samples.

			Number of NGS-positive pools (number of reads)										
Sample types	Number of samples	Number of pools	*Anaplasma* spp.	*Bartonella* spp.	*Borrelia* spp.	*Coxiella* spp.	*Ehrlichia* spp.	*Ca.* Neoehrlichia spp.	*Francisella* spp.	*Leptospira* spp.	*O. tsutsugamushi*	*Rickettsia* spp.	*Neorickettsia* spp.
UFI patients	200	23	8	2	0	4	0	0	0	3	9	0	0
			(14–13,226)	(239–7,797)		(18–347)				(10–4,393)	(224–7,347)		
Rodents	309	64	20	47	10	0	9	11	0	3	2	3	2
			(13–14,357)	(18–60,351)	(11–1,384)		(21–53,134)	(17–5,141)		(1,771–11,216)	(92–212)	(31–150)	(51–2,803)
Rodent chiggers	199	43	0	2	8	1	0	0	4	2	8	0	0
				(23–808)	(26–2,965)	(15)			(28–1,713)	(19–135)	(10–974)		
Rodent ticks	59	17	5	6	2	7	1	0	6	1	0	10	0
			(13–15,237)	(68–7,624)	(90–264)	(30–65,264)	(14,725)		(16–13,144)	(36)		(20–23,924)	
Rodent fleas	23	8	0	5	0	0	0	0	0	0	0	0	0
				(35–119,256)									
Rodent lice	4	4	0	4	0	0	0	0	0	0	0	1	0
				(3,834–90,081)								(1,366)	
Mammal ticks	35	8	4	0	0	7	0	0	1	0	0	4	0
			(367–10,042)			(264–35,079)			(6,960)			(12–2,074)	
Mammal fleas	88	12	0	3	0	2	0	0	0	0	0	12	0
				(19–1,001)		(6,053–39,729)						(11–22,961)	
Mammal lice	12	4	0	0	0	0	0	0	0	0	0	0	0

*Numbers of NGS-positive pools in each sample type are shown for 11 bacterial genera and the number of reads indicated by range are provided in parentheses. The pooling strategy for each sample type was provided in Supplementary Table S2.*

### Microbial Profiling in Human, Rodent, and Vector Populations

**FIGURE 3:**
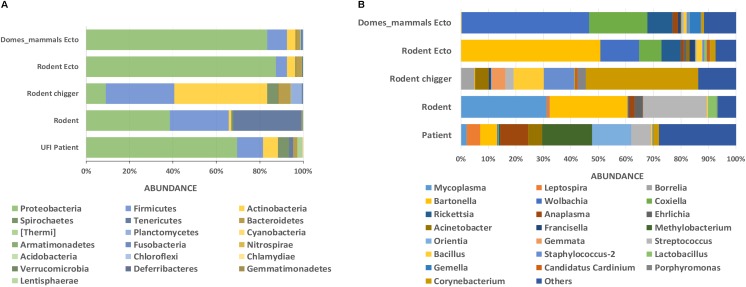
Taxonomic diversity and relative abundance at the phylum **(A)** and genus **(B)** level of bacterial community in UFI patients, rodents, chiggers, and ectoparasites collected from rodents, and ectoparasites collected from domesticated mammals. Phyla were identified on the basis of a confidence threshold cutoff of 77%, and genera on a confidence threshold cutoff of >90% using the Green Genes reference database. The percent relative abundances are of the total number of OTUs. Color legend for each phylum **(A)** or genus **(B)** was indicated below the bar graph.

The classification of OTUs from each sample were made against Greengenes reference databases, with SILVA reference databases as a secondary database, and the similarity confidence threshold for each taxonomic level was set at 0.97 in CLC microbial genomic module. There were 19 recorded phyla found among all sample types and the ten most abundant phyla were seen in all sample types (Proteobacteria, Firmicutes, Actinobacteria, Spirochaetes, Tenericutes, Bacteroidetes, [Thermi], Planctomycetes, Fusobacteria, Cyanobacteria) (Figure 3A). The most prevalent phylum in all sample types was Proteobacteria and it was most abundant in ectoparasites from rodents (88.0%), domesticated mammals (83.0%), and UFI patients (70.0%) (Figure 3A). In rodent populations, the phyla Proteobacteria, Tenericutes, and Firmicutes were most abundant (38.0, 31.0, and 27.0%, respectively). In chiggers, the highest abundance was of Actinobacteria (43.0%), followed by Firmicutes (32.0%), and Proteobacteria (9.0%). Other interesting phyla include Spirochaetes found in UFI patients (5%), chiggers (5%), and rodents (1%); and two phyla, Thermi and Plantomycetes, that were found most commonly in UFI patients and chigger samples, respectively.

At the genus level, as many as 651 genera were found among sample populations with the greatest diversity of taxa belonging to the phylum Proteobacteria (*n* = 254 genera), followed by Actinobacteria (*n* = 152), Firmicutes (*n* = 122), and Bacteroidetes (*n* = 57). The most abundant reads belonged to genera in Proteobacteria from UFI patients (*Methylobacterium* = 18%, *Orientia* = 14%, *Anaplasma* = 10%), rodent associated ectoparasites (*Bartonella* = 50%, *Wolbachia* = 14%, *Coxiella* = 8%, and *Rickettsia* = 7%), and ectoparasites collected from domesticated mammals (*Wolbachia* = 46%, *Coxiella* = 21%, and *Rickettsia* = 9%) (Figure 3B). In rodent samples, the most abundant bacterial genera were distributed among three major phyla; Tenericutes (*Mycoplasma* = 31%), Proteobacteria (*Bartonella* = 28%), and Firmicutes (*Streptococcus* = 23%). With the chigger samples, the majority of reads belonged to *Corynebacterium* spp. in the Actinobacteria phylum (41%), followed by *Bacillus* spp. and *Staphylococcus* spp. in the Firmicutes phylum (both with 11% abundance).

### Bacterial Endosymbionts in Ectoparasites From Rodents and Domesticated Mammals

*Wolbachia* spp. was found mainly in mammal fleas (*n* = 10 NGS pools) with one pool each in rodents and ticks, lice, and fleas collected from rodents. The data of endosymbionts detected in NGS pools were provided as online Supplementary Data (Supplementary Figure S1). Few reads were detected in UFI patients (273 reads). This may have been due to cross-contamination during the sample preparation process. *Coxiella* endosymbionts were equally found in ticks collected from rodents and domesticated mammals. Few (*n* = 2) were associated with *Francisella* spp., and they were suspected as endosymbionts since they tested negative by a confirmatory assay (qPCR and PCR). Only one NGS pool of chiggers was found to carry a *Coxiella* endosymbiont. *Francisella* endosymbiont was mainly found in chiggers and ticks collected from rodents but one pool was also found in ticks from a dog. *Candidatus* Cardinium was mostly detected in chiggers; however, it was also detected in one pool of rodent fleas and in one pool of rodent ticks. *Rickettsia* spp. were mainly detected in ticks and lice from rodents and fleas and ticks from domesticated mammals. However, some *Rickettsia* were pathogenic species as confirmed by real-time PCR and DNA sequencing. This phenomenon was mostly observed in *Rickettsia* from flea pools (mostly from domesticated mammals) where *Rickettsia* could be identified to species and excluded from being identified as endosymbiont bacteria. Therefore, *Rickettsia* endosymbiont was not discussed here. When considering only known and confirmed endosymbionts (*Wolbachia*, *Coxiella*, and *Ca. Cardinium*) found in vectors, there was usually one or two predominant endosymbionts harbored by each vector type.

### Detection of Pathogenic Bacteria in all Populations by NGS Results and Prevalence Rate After Verification by Confirmatory Assays

After NGS analysis, eleven potential pathogenic bacteria were detected among samples. Table 2 shows details about positive NGS pools and the number of reads indicated by range for each potential pathogen detected among sample populations. *Bartonella* spp. were the most prevalent and detectable bacteria among samples studied and the highest infection rate was found in rodent population (47/64 NGS pools). *Bartonella* spp. were found in most sample types with the exception of ticks and lice from domesticated mammals (69/183 NGS pools). The second most prevalent bacteria was *Anaplasma* spp. (37/183 NGS pools) which was found in UFI patients (8/23), rodents (20/64) and rodent-associated ticks (5/17), and ticks collected from domesticated mammals (4/8). *Rickettsia* spp. were also found in high prevalence as well (30/183), mostly in pools of fleas collected from domesticated mammals (12/12), 10 out of 17 pools of rodent ticks, 4/8 pools of ticks from domesticated mammals, 3/64 pools of rodents, and 1/4 pools of rodent lice. *Borrelia* spp., *Coxiella* spp., and *Orientia* spp. were equally detected; however, the distribution among populations was less similar. *Borrelia* spp. was found among rodents and its ectoparasites (chiggers and ticks), while *Orientia* spp. was detected among UFI patients, rodents, and chigger populations. *Coxiella* spp. were detected in a wide range of populations such as UFI patients, chiggers and ticks collected from rodents, and ticks and fleas collected from domesticated mammals. Other less prevalent pathogens such as *Ehrlichia* spp. (Rodents and their ticks), *Candidatus* Neoehrlichia spp. (rodents), *Francisella* spp. (chiggers and ticks from rodents, ticks from domesticated mammals), *Leptospira* spp. (UFI patients, rodents and their associated chiggers and ticks), and *Neorickettsia* spp. (rodents) were also detected in 2–11 out of 183 pools. Rodents carried the most diverse and wide-range of potential pathogenic bacteria and as many as 9 genera were detected. Likewise, ticks collected from rodents had the greatest pathogenic bacterial diversity of any vectors sampled (8 genera).

**FIGURE 4:**
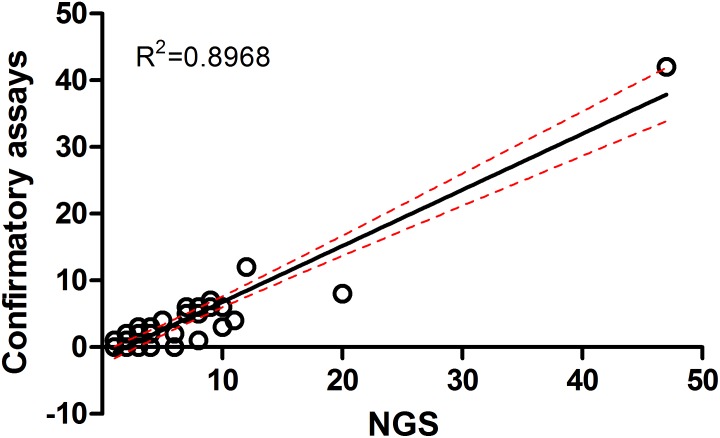
Correlation of metagenomic sequencing with confirmatory assays using qPCR and PCR methods for detection of bacterial pathogens in a variety of samples.

In this study, all samples present in each positive NGS pool were individually tested by confirmatory assays using either real-time PCR or conventional PCR (Supplementary Table S1). All amplified products were sequenced by the Sanger method. The correlation between the number of NGS-positive pools and the number of positive pools verified by confirmatory assays was determined using linear regression analysis (Figure 4). The results from both assays were positively correlated with *R*^2^ = 0.8968 (95% confidence interval = 0.7440–0.9368) or *R*^2^ = 0.6004 (95% confidence interval = 0.3950–0.7024) when the far point was removed. Prevalence rates for each pathogen were calculated based on NGS results verified by confirmatory assays or a combination of both NGS results and routine screening tests as mentioned earlier. Table 3 shows the prevalence rate for each pathogen detected among samples. A high prevalence of *Bartonella* spp. was seen in rodents, rodent fleas, and rodent lice populations (41.1, 65.2, and 75.0%, respectively). The prevalence of *Rickettsia* spp. was highest in fleas collected from domesticated mammals, mostly from dogs (84.1%), followed by rodent lice (25.0%), and rodent ticks (6.8%). *Coxiella* spp. was detected at highest prevalence in ticks collected from rodents and domesticated mammals (32.2 and 71.4%), but later was identified as a *Coxiella* endosymbiont (Table 4). Other highly pathogenic species known to cause disease in humans and animals were detected among vectors, rodents, and UFI patient samples, although at low prevalence. These included *O. tsutsugamushi*, *Anaplasma* spp., *Borrelia* spp., and *Leptospira* spp.

**Table 3:** Prevalence of pathogenic bacteria detected in sample populations.

		NGS analysis	Confirmatory assays	
Sample types	Detected pathogens (genera)	Number of NGS-positive pools (number of samples tested)	Number of positive pools (number positive samples)	Number positive/total number of samples studied (% prevalence)
UFI patients	*Anaplasma* spp.	8 (66)	1 (1)	1/200 (0.5)
	*Bartonella* spp.	2 (21)	1 (1)	1/200 (0.5)
	*Coxiella* spp.	4 (41)	0 (0)	0/200 (0)
	*Leptospira* spp.	3 (32)	0 (0)	0/200 (0)
	*Orientia* spp.	9 (78)	7 (11)	11/200 (5.5)
Rodents	*Anaplasma* spp.	20 (99)	8 (9)	9/309 (2.9)
	*Bartonella* spp.	47 (259)	42 (127)	127/309 (41.1)
	*Borrelia* spp.	10 (64)	6 (10)	10/309 (3.2)
	*Ehrlichia* spp.	9 (47)	6 (6)	6/309 (1.9)
	*Candidatus* Neoehrlichia spp.	11 (64)	4 (4)	4/309 (1.3)
	*Leptospira* spp.	3 (16)	3 (4)	4/309 (1.3)
	*Orientia* spp.	2 (5)	0	3/309 (1.0)^∗^
	*Rickettsia* spp.	3 (11)	0 (0)	0/309 (0)
	*Neorickettsia* spp.	2 (17)	2 (2)	2/309 (0.7)
Rodent chiggers	*Bartonella* spp.	2 (20)	0 (0)	0/199 (0)
	*Borrelia* spp.	8 (40)	5 (7)	7/199 (3.6)
	*Coxiella* spp.	1 (4)	1 (1)	1/199 (0.5)
	*Francisella* spp.	4 (18)	0 (0)	0/199 (0)
	*Leptospira* spp.	2 (16)	0 (0)	0/199 (0)
	*Orientia* spp.	8 (32)	6 (6)	6/199 (3.0)
Rodent ticks	*Anaplasma* spp.	5 (12)	4 (4)	4/59 (6.8)
	*Bartonella* spp.	6 (18)	2 (2)	2/59 (3.4)
	*Borrelia* spp.	2 (2)	2 (2)	2/59 (3.4)
	*Coxiella* spp.	7 (40)	5 (19)	19/59 (32.2)
	*Ehrlichia* spp.	1 (1)	1 (1)	1/59 (1.7)
	*Francisella* spp.	6 (26)	0 (0)	0/59 (0)
	*Leptospira* spp.	1 (1)	0 (0)	0/59 (0)
	*Rickettsia* spp.	10 (43)	3 (4)	4/59 (6.8)
Rodent fleas	*Bartonella* spp.	5 (16)	4 (15)	15/23 (65.2)
Rodent lice	*Bartonella* spp.	4 (4)	3 (3)	3/4 (75.0)
	*Rickettsia* spp.	1 (1)	1 (1)	1/4 (25.0)
Tick	*Anaplasma* spp.	4 (21)	2 (4)	4/35 (11.4)
	*Coxiella* spp.	7 (34)	6 (25)	25/35 (71.4)
	*Francisella* spp.	1 (4)	0 (0)	0/35 (0)
	*Rickettsia* spp.	4 (30)	0 (0)	0/35 (0)
Flea	*Bartonella* spp.	3 (21)	2 (5)	7/88 (7.9)^∗^
	*Coxiella* spp.	2 (2)	0 (0)	0/88 (0)
	*Rickettsia* spp.	12 (88)	12 (74)	74/88 (84.1)

*The NGS results verified by conventional methods (confirmatory assays) are shown as well as the comparison of the number of positive pools performed by both assays. ^∗^Numbers of positive samples are from the results of both NGS and routine screening in which all samples were screened by real-time PCR or conventional PCR.*

**Table 4:** Pathogen characterization by DNA sequence and phylogenetic analyses.

Sample type	Host	Pathogens	Number of positive	Number of characterization	Target gene(s)	Number of sequences match (% identity)
UFI patient	N/A	*Anaplasma* spp.	1	1	groEL	1×*Anaplasma* spp. (96.8)
		*Bartonella* spp.	1	1	gltA	1×*B. quintana* (100)
		*Orientia* spp.	11	11	56 kDa TSA	2×*O. tsutsugamushi*_Karp A genotype (99.6), 7×*O. tsutsugamushi*_JG-C genotype (98.7–99.1), 1x *O. tsutsugamushi*_Kato B genotype (90.1), 1x *O. tsutsugamushi*_unknown genotype (73.2)
Rodents	N/A	*Anaplasma* spp.	9	9	16S	6×*A. bovis* (97.3–100), 3×*A. phagocytophilum* (100)
		*Bartonella* spp.	127	62	ssrA/gltA/nuoG	6×*B. elizabethae* (ssrA:99.1–99.5), 3×*B. japonica* (ssrA:99.5), 3×*B. queenslandensis* (gltA: 97.2), 1×*B. silvatica* (ssrA: 99.1), 1×*Candidatus* B. thailandensis (gltA: 98.1), 49×*Bartonella* spp. (ssrA: 96.4–98.6/nuoG: 90.2–98.1/gltA:96.3)
		*Borrelia* spp.	10	2	fla/16S	1×*Bor. yangtzensis* (16S: 98.4), 1×*Bor. miyamotoi* (flaB: 99.6/16S: 99.7)
		*Ehrlichia/Ca.* Neoehrlichia spp.	10	6	16S	2×*Ehrlichia* spp. (97.9–100), 4×*Candidatus* Neoehrlichia mikurensis (96.4–99.2),
		*Leptospira* spp.	4	4	16S/secY	4×*L. interrogans* (16S: 99.8–100/secY: 98.0)
		*Neorickettsia* spp.	2	2	16S	2×*Neorickettsia* spp. (85.7–100)
		*Orientia* spp.	0	3^∗^	56 kDa TSA	1×*O. tsutsugamush*i_Saitama-karp genotype (91.6), 1×*O. tsutsugamushi*_JG-C genotype (98.7), 1×*O. tsutsugamushi*_TA763 B genotype (99.3)
Chiggers	Rodents	*Borrelia* spp.	7	5	16S/flaB	2×*Borrelia* spp. (16S: 97.4/flaB: 68.0–68.3)
					flaB	3×*Borrelia* spp. (68.3–79.2)
		*Coxiella* spp.	1	1	16S	1×*Coxiella* endosymbiont (98.2)
		*Orientia* spp.	6	6	56 kDa TSA	1×*O. tsutsugamushi*_TA763 B genotypes (97.9), 1×*O. tsutsugamushi*_Kato B genotype (76.7), 3×*O. tsutsugamushi*_Saitama-Karp genotype (92.1–93.1), 1×*O. tsutsugamushi*_Karp A (99.6)
Ticks	Rodents	*Anaplasma* spp.	4	4	16S	2×*A. bovis* (100), 2×*A. phagocytophilum* (100)
		*Ehrlichia* spp.	1	1	16S/groEL	1×*Ehrlichia* spp. (16S: 99.6, groEL: 87.7)
		*Borrelia* spp.	2	2	flaB	2×*Bor. yangtzensis* (97.6–99.3)
		*Coxiella* spp.	19	19	16S	19×*Coxiella* endosymbiont (97.4–100)
		*Rickettsia* spp.	4	4	gltA	3×*Candidatus* Rickettsia jingxinensis (99.6), 1×*Rickettsia* spp. (99.4)
Fleas	Rodents	*Bartonella* spp.	15	3	ssrA/nuoG	1×*B. silvatica* (ssrA: 99.1), 2×*Bartonella* spp. (nuoG: 77.5–91.4)
Lice	Rodents	*Bartonella* spp.	3	2	gltA/nuoG	1×*B. queenslandensis* (gltA: 97.2), 1×*Bartonella* spp. (nuoG: 91.1)
		*Rickettsia* spp.	1	1	gltA	1×*R. asemboensis* (100)
Ticks	Domesticated mammals	*Anaplasma* spp.	4	3	16S/groEL	2×*A. platys* (16S/groEL: 100), 1×*A. bovis* (16S: 99.2)
		*Coxiella* spp.	25	25	16S	25×*Coxiella* endosymbionts (93.9–99.4)
Fleas	Domesticated mammals	*Bartonella* spp.	5	7^∗^	ssrA/gltA	5×*B. clarridgeiae* (gltA/ssrA: 100), 2×*B. elizabethae* (gltA: 96.4)
		*Rickettsia* spp.	74	8	gltA	7×*R. asemboensis* (100), 1×*Candidatus* Rickettsia senegalensis (100)

*^∗^Number of positive samples is from the results of both NGS and routine screening; N/A, not available.*

*Orientia tsutsugamushi* was found among UFI patients (11/200, 5.5%), chiggers (6/199, 3.0%) (a well-known scrub typhus vector), and rodents (3/309, 1.0%). *Anaplasma* spp. were also found in one UFI patient (1/200, 0.5%), rodents (9/309, 2.9%) and ticks collected from rodents (4/59, 6.8%) and domesticated mammals (4/35, 11.4%), while *Borrelia* spp. were found only in rodents and their associated ticks and chiggers with prevalence rates ranging from 3.2 to 3.6%. Other bacteria such as *Leptospira* spp., *Ehrlichia* spp., *Candidatus* Neoehrlichia spp., and *Neorickettsia* spp. were found in rodents and their associated ticks with prevalence rates in the range of 0.7–1.7%.

### Characterization of Bacterial Species by DNA Sequence (Sanger Sequencing) and Phylogenetic Analyses

**FIGURE 5:**
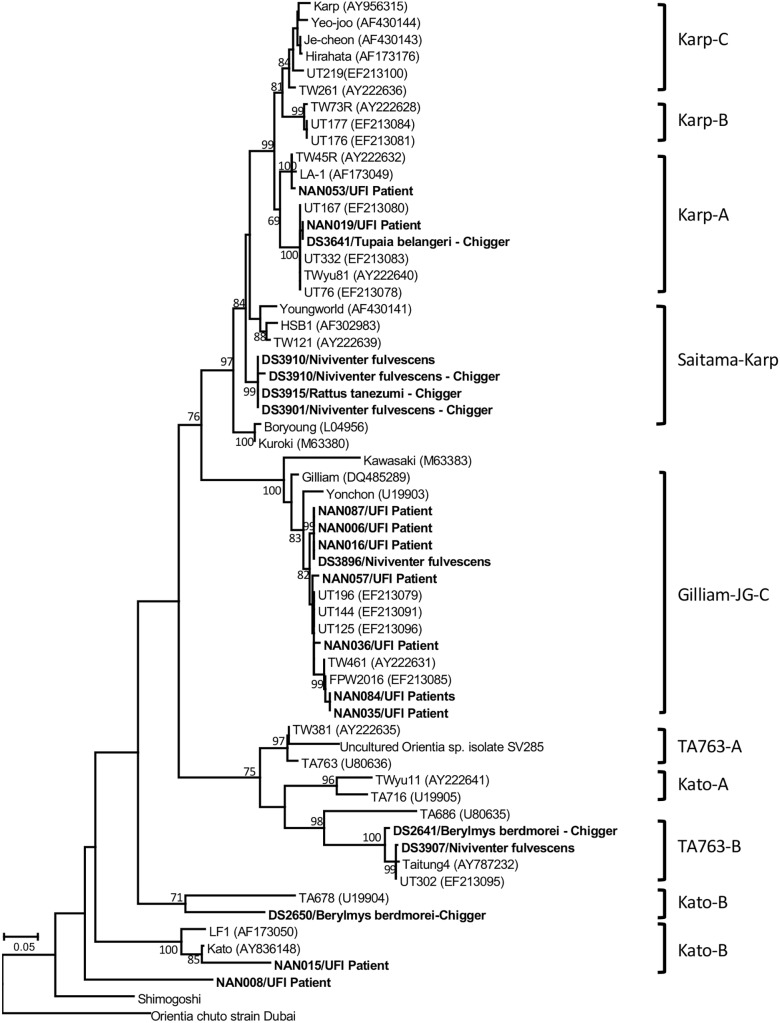
Phylogenetic tree analysis of *O. tsutsugamushi* genotypes detected among UFI patients, rodents, and chiggers in Nan province, Thailand (indicated in bold letters). A maximum likelihood tree was constructed based on 56-kDa type-specific antigen gene sequences using the GTR+G model of nucleotide substitution in the MEGA 6 program with bootstrapping (1000 replicates).

Several bacterial species were identified based on their highest similarity to reference sequences (GenBank database) as shown in Table 4, as well as their identity (%) corresponding to highly matched reference sequences as indicated in parenthesis after each pathogen. Only some important pathogenic bacteria are discussed here. *O. tsutsugamushi* and *Leptospira* spp. are well-known pathogenic bacteria endemic to Thailand and were frequently detected in the samples studied. *O. tsutsugamushi* was detected among UFI patients, rodents, and chiggers and it was observed that some populations shared the same genotypes as demonstrated by the phylogenetic analysis present in Figure 5. Although *L. interrogans* was confirmed to be present in rodent population, the pathogenic status of *Leptospira* spp. (NGS reads in the range 36–4,393) belonging to other populations could not be verified (Tables 3, 4). *B. quintana*, the causative agent of trench fever, was detected in one UFI patient but not in other populations. However, other common rodent-associated *Bartonella* species were found in rodents and their associated fleas and lice. Interestingly, *B. clarridgeiae*, a possible causative agent of cat-scratch disease, was found in 2 pools of fleas (*Ctenocephalides felis*) collected from domesticated mammals (dogs). Human granulocytic anaplasmosis (*A. phagocytophilum*) was also detected in rodents and their associated *Ixodes* ticks. Other anaplasmosis causative agents, *A. bovis* and *A. platys* were also detected from rodents and their ticks, and ticks of domesticated mammals. Interestingly, one UFI patient was positive for *Anaplasma* spp. with 96.8% identity to Uncultured *Anaplasma* spp. detected in a tick from China (GenBank accession No. KF728361.1). Even though its identity to pathogenic species, *A. phagocytophilum* and *A. bovis*, was 92.3–92.7%, the phylogenetic relationship of its *gro*EL sequence with these pathogenic species was relatively closer than other known species in the tree (online Supplementary Data). *Borrelia miyamotoi* was detected in one rodent (*Niviventer* spp.) with 99.6% identity to reference sequence of flagellin and 16S rRNA genes. *Borrelia yangtzensis*, a newly recognized *B. valaisiana*-like strain, was also found in one rodent and 2 tick pools collected from rodents (*Ixodes* spp.). *Borrelia* spp. detected in chiggers were sequenced and the results showed that only 2 sequences (16S rRNA gene) were highly similar to *Borrelia* species (97.4%) which were not grouped in any of 2 *Borrelia* groups; relapsing fever and Lyme. All five *fla*B sequences had very low identity to *Borrelia* species (68.0–79.2%) and were very distantly related to the genus of *Borrelia* as demonstrated by the phylogenetic tree analysis. The phylogenetic trees for most of pathogens detected were provided as online Supplementary Data.

### Seroprevalence in UFI Patients for Selected Pathogens With Highest Prevalence in Rodent and Vector Populations

**FIGURE 6:**
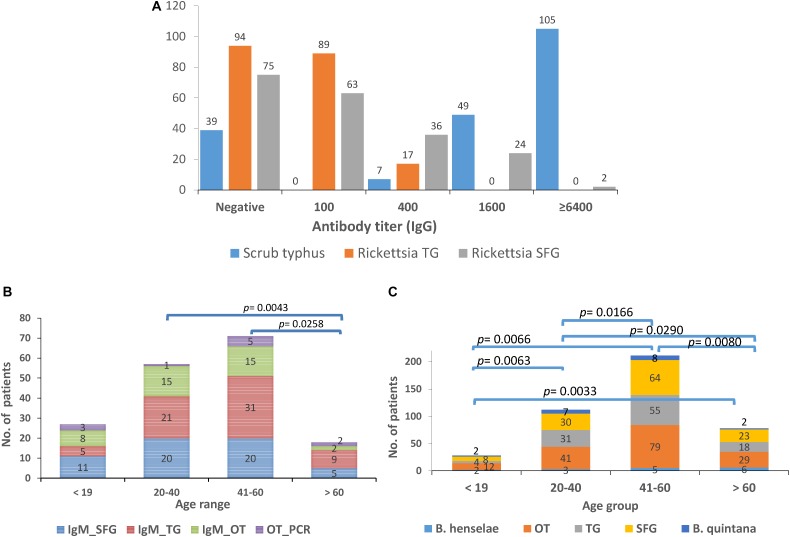
Seroprevalence (IgM and IgG) of scrub typhus [*O. tsutsugamushi* (OT)], and rickettsiosis in UFI patients from Bo Kluea hospital, Nan province. The IgG titers for scrub typhus and rickettsiosis [typhus group (TG) and spotted fever group (SFG)] are shown **(A)** as well as the seroprevalence of IgM **(B)** and IgG **(C)** antibodies among age groups (<19, 20–40, 41–60, >60 years old).

The seroprevalence of the most prevalent pathogenic bacteria was determined in UFI patient sera (*n* = 200). IFA and ELISA assays were performed to examine the presence of IgM or IgG antibodies and to measure the titer of IgG antibody in UFI patients (Table 5). The results of IFA assays testing for IgM antibodies against scrub typhus, murine typhus and spotted fever group *Rickettsia* (SFGR) showed relatively high numbers of patients with past infection. Patients with IgM antibody against murine typhus had the highest number accounting for 33.0% (66/200), followed by SFGR (28.0%, 56/200), and scrub typhus (20.0%, 40/200). However, when the same set of sera (*n* = 200) were tested for their IgG titer, scrub typhus seems to dominate the other two diseases with 154 patients having higher titers (1,600 and >6,400), compared to SFGR (*n* = 26) or murine typhus (*n* = 0) (Table 5 and Figure 6A). Cross-reactivity between scrub typhus and rickettsiosis was determined using positive controls from commercial kits and the results showed no cross-reactivity was found among these pathogens for IgG and IgM.

**Table 5:** Seroprevalence of scrub typhus, *Rickettsia* typhus group, and *Rickettsia* spotted fever group in UFI patients, Nan province, Thailand.

					Antibody titer			
Pathogens	Assay types	Antibody types	Screening titer	Number of positive (% prevalence)	ELISA/IFA (100/32)	ELISA/IFA (400/64)	ELISA/IFA (1600/128)	ELISA/IFA (>6400/256)
*Orientia tsutsugamushi*	IFA	IgM	1:64	40 (20.0)	–	–	–	–
*Orientia tsutsugamushi*	ELISA	IgG	1:100	161 (80.5)	0	7	49	105
*Rickettsia* spp., typhus group	IFA	IgM	1:64	66 (33.0)	–	–	–	–
*Rickettsia* spp., typhus group	ELISA	IgG	1:100	108 (54.0)	91	17	0	0
*Rickettsia* spp., spotted fever group	IFA	IgM	1:64	56 (28.0)	–	–	–	–
*Rickettsia* spp., spotted fever group	ELISA	IgG	1:100	125 (62.5)	63	36	24	2
*Bartonella quintana*	IFA	IgG	1:32	19 (9.5)	2	12	3	2
*Bartonella henselae*	IFA	IgG	1:64	16 (8.0)	–	–	–	–
*Anaplasma phagocytophilum*	IFA	IgG	1:80	1 (0.5)	–	–	–	–

*Seroprevalence of other pathogens (cat-scratch disease, trench Fever, anaplasmosis) detected by NGS approach are also shown.*

Patients were grouped into four age groups; <19, 20–40, 41–60, and >60 years old, and by sex; male and female, to determine how seroprevalence to rickettsiosis and scrub typhus differed among the groups. The data showed higher seroprevalence (both IgM and IgG) of the two diseases in two age groups (20–40 and 41–60 years old) compared to the other two age groups (<19 and >60) with statistical significance (Figure 6B,C). However, the overall prevalence was not different between male and female patients. In addition, lower seroprevalence (IgG) was observed for other pathogens such as *B. quintana* (9.5%, 19/200), *B. henselae* (8.0%, 16/200), and *A. phagocytophilum* (0.5%, 1/200) (Table 5).

### Circulation of Pathogenic Bacteria Among Human, Reservoir Host, and Vector Populations

**FIGURE 7:**
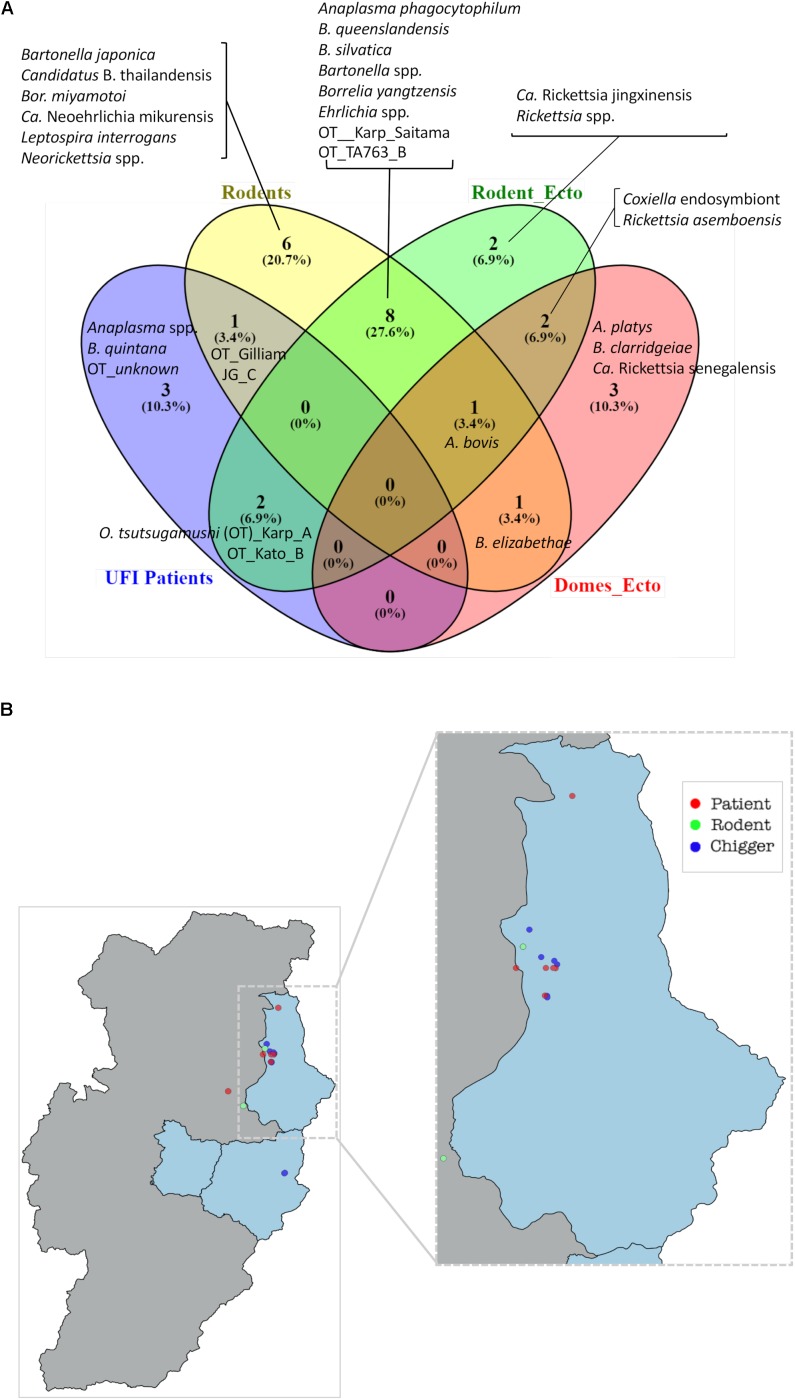
Venn diagram (Oliveros et al., 2007–2015) indicates the bacteria species shared between populations or unique to each of them **(A)**. Bacterial species were identified on the basis of DNA sequence and phylogenetic analyses of their target genes (Supplementary Table S1). Rodent_ecto = ectoparasites (chiggers, ticks, fleas, and lice) collected from rodents, Domes_ecto = ectoparasites collected from domesticated mammals. Map of *O. tsutsugamushi*-positive samples in Bo Kluea district, Nan **(B)**. Each dot represents only positive samples found among UFI patients (red), chigger pools (blue), and rodent populations (green).

A Venn diagram illustrates the shared bacterial species among samples (Figure 7A). Samples were grouped into four groups [UFI patients, rodents, rodent-associated ectoparasites (including chiggers)], and ectoparasites collected from domesticated mammals. The diagram illustrates the sharing of *O. tsutsugamushi* genotypes among UFI patients, chiggers, and rodent populations. Coordinates of all positive samples for *O. tsutsugamushi* were mapped and it was shown that almost all positive samples clustered together in Bo Kluea district (Figure 7B). *A. bovis* was another pathogen found circulating among animal reservoirs and ticks. The pathogen was detected in *Rattus* rats and *Tupaia glis* (common treeshrew) as well as in *Rhipicephalus sanguineus* and *Haemaphysalis bandictota* ticks where both tick species were known to share common hosts (*Bandicota indica* and *Rattus* rats) (Tanskul et al., 1983). Other pathogens such as *A. phagocytophilum*, *Borrelia yanzensis*, and various *O. tsutsugamushi* genotypes were shared between rodents and their associated vectors and are shown in Figure 7A. Pathogens solely detected in each population are also indicated in the figure, especially *Borrelia miyamotoi*, *B. clarridgeiae*, and *Leptospirosis interrogans* which are known to cause infections in humans. *A. platys* was found from *R. sanguineus* ticks collected from a dog and is the causative agent of canine ehrlichiosis.

**FIGURE 8:**
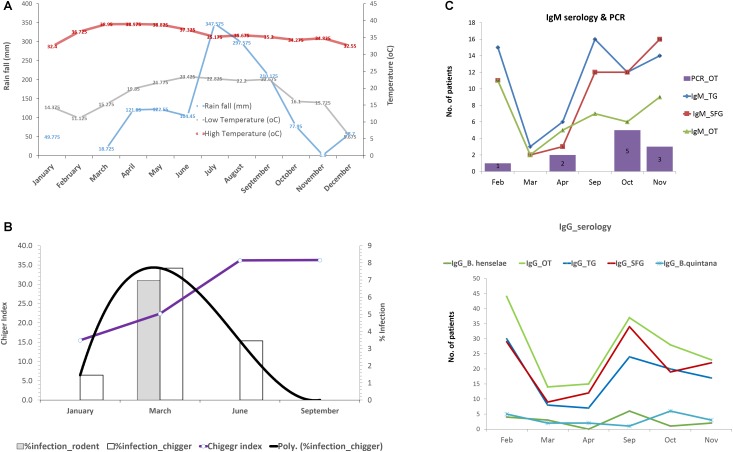
Rainfall (mm) and temperature (°C) during the year 2017 in Nan province **(A)**. Infection rate of *O. tsutsugamushi* in rodents and chigger pools as well as the chigger index recovered from rodents during wet (April–September) and dry (October–March) seasons **(B)**. Seroprevalence (IgM and IgG) of scrub typhus, rickettsiosis, and *O. tsutsugamushi* detection in UFI patients were plotted according to the time of collection from Bo Kluea hospital, Nan **(C)**.

The effect of environmental factors on the prevalence and transmission of scrub typhus among populations studied was also evaluated. Rainfall (mm) and temperature in 2017 from Nan province was acquired from the Thai Meteorological Department^4^. The rainy season started from late April through September corresponding to increased rainfall (mm) recorded during this period of the year (Figure 8A). The temperature was relatively constant throughout the year except a slight decrease at the end and the beginning of the year (October–March). The chigger index (no. of chigger/number of hosts collected) and the *O. tsutsugamushi* infection rates in rodents and chiggers were examined each month (Figure 8B). The chigger index slightly increased at the beginning of the year and peaked around June–September. *O. tsutsugamushi* in chigger could be found almost every month and the highest infection rates were in March. On the other hand, the number of UFI patients with IgM antibody against scrub typhus slowly increased from April to October and peaked at the end through the beginning of the year (November–February) (Figure 8C). *O. tsutsugamushi* was also detected by PCR from patient whole blood samples but the number did not correlate well with the number of patients having IgM seroactivity. Interestingly, the number of patients with rickettsiosis IgM increased sharply from April to September and this high number continued through the rest of the year. Likewise, the same pattern was observed with their corresponding IgGs although the number of patients with scrub typhus IgG and its titer seemed to be higher than that observed for rickettsiosis (TG and SFG) (Figure 8C and Table 5).

## Discussion

Scrub typhus is a major public health problem in Nan province with the highest cases of scrub typhus infection (152.64 per 100,000 population) reported to Bureau of Epidemiology, Department of Disease Control, the Ministry of Public Health (MoPH), Thailand in 2017^5^. Samples analyzed in this study included all factors/populations that are involved in disease transmission. Most samples from UFI patients were collected from Bo Kluea hospital in 2017, while rodents and ectoparasites were collected twice a year (dry and wet seasons) from 2014 until the beginning of 2018 at field collection sites in Mae Charim, Phu Phiang, and Bo Kluea districts.

In this study, extraction and PCR controls were included in each NGS run to exclude bacteria genera commonly found in molecular reagents, water, and other environments (Tanner et al., 1998; Goodrich et al., 2014). Several common bacterial genera were found in controls similar to previous studies (Salter et al., 2014; Razzauti et al., 2015). Some potential zoonotic and pathogenic bacteria were also detected in controls such as *Bartonella* spp., *Leptospira* spp., and *Rickettsia* spp. albeit at relatively low numbers of reads. Therefore, we applied cut-off values (number of reads detected in controls) for those bacteria detected in controls and applied these numbers to all samples in our study. Contamination likely came from cross-contamination during sample processing and carry-over between sequencing runs (Swei et al., 2013). The NGS technique has many benefits over conventional tests since it does not require prior knowledge of the target pathogens which conventional tests most often rely upon. However, there is no standardized protocol for all laboratories and contamination from reagents and the environment can complicate the analysis. Therefore, conventional methods were also employed as confirmatory assays in this study. The 16S sequence can only discriminate pathogens to the genus level with confidence. However, one genus may consist of multiple species, some of which may not be pathogenic to humans or animals. Therefore, it is necessary to further identify the bacteria genera detected by NGS to the species level using conventional PCR or Sanger sequencing.

Several bacterial genera are saprophytes and commensals or can be found as contaminants in reagents and the environment. Therefore, they are considered non-pathogenic bacteria and were not considered in our analysis (Razzauti et al., 2015). A list of bacteria commonly detected in reagents and laboratory contamination was previously published by Salter et al. (2014). In this study, most of the bacterial genera found in sample populations were commensals or saprophytes such as *Methylobacterium* in UFI patients, *Mycoplasma* and *Streptococcus* in rodents, and *Corynebacterium* in chiggers. These bacteria comprised 18–41% of the total OTU reads in each population. Detection of bacterial DNA in human blood was not unexpected since healthy blood donors also contain bacterial DNA such as Proteobacteria (>80%), Actinobacteria, Firmicutes, and Bacteroidetes (Paisse et al., 2016) which is similar to what we found in UFI patients. Bacterial endosymbionts were highly abundant in ectoparasites such as *Wolbachia* (46% in fleas from dogs) or *Coxiella* endosymbionts in ticks from rodents and dogs. What we found in this study is that fleas and ticks collected from domesticated mammals harbored one predominant endosymbiont such as *Wolbachia* in fleas (*Ctenocephalides felis*) and *Coxiella* endosymbiont in *R. sanguineus* ticks collected from dogs. Chiggers and ticks collected from rodents had two predominant endosymbionts such as *Haemaphysalis* ticks which carried both *Coxiella* and *Francisella* endosymbionts, while chiggers had both *Candidatus* Cardinium and *Francisella* endosymbionts. However, since ectoparasites were pooled before the NGS procedure, the number of endosymbionts or co-infection of endosymbionts in single vectors could not be determined.

In this study, a high prevalence from NGS results was observed for few bacterial genera; however, some genera could not be verified with conventional assays such as real-time PCR, PCR, or DNA sequencing. The main reason was likely that the number of reads was too low and below the limit of detection for the conventional test, or they could have been non-pathogenic strains or species and were not picked up by confirmatory assays. For example, *Leptospira* spp. comprise both pathogenic, intermediate, and saprophytic (non-pathogenic) species that can be introduced as contaminants from the environment into samples. Here *Leptospira* spp. were only detected in the rodent population, although NGS analysis showed reads were also detected in UFI patients, chiggers, and ticks at lower levels. However, testing with confirmatory assay resulted in no signal or PCR product using a genus-based assay (Ahmed et al., 2009) with primer sets targeting house-keeping genes such as *gyr*B or *Sec*Y (Slack et al., 2006; Victoria et al., 2008). In some cases, PCR assays targeting *Leptospira* 16S rRNA genes showed some positive bands for UFI patients but the product size was shorter than expected and DNA sequences from these products matched only human DNA (100%). *Rickettsia* spp. and *Francisella* spp. could not be verified in some sample types such as ticks, chiggers, and rodents. These could be endosymbiont bacteria which our assays could not detect (Wright et al., 2011; Takhampunya et al., 2017).

Originally, the confirmatory assays did not verify the NGS results (2 pools) of *O. tsutsugamushi* detected in rodents. However, since scrub typhus detection has been run in our lab as part of routine surveillance assays, three *O. tsutsugamushi*-positive rodents were verified by a routine real-time PCR test (Table 4). Similarly, four *Bartonella* species detected in fleas from domesticated mammals were also verified by a routine real-time PCR test and they were included in Table 4. NGS seems to have less sensitivity than the conventional method. In support of this observation, a previous study has compared MiSeq and RNA-seq, and found that MiSeq cannot detect bacteria at a value lower than 4% prevalence in the population and thus RNA-seq is better in terms of sensitivity (Razzauti et al., 2015). In all likelihood, this is due to differences in sequencing depth for each of the techniques used. The detection of *B. quintana* in one UFI patient and *B. clarridgeiae* in *Ctenocephalides felis* fleas provides significant evidence that Trench fever and cat-scratch disease-causing bacteria are present in the study area. The seroprevalence data (IgG) of *B. quintana* and *B. henselae* also confirmed previous human exposure to these bacteria. Although we detected *A. phagocytophilum* (anaplasmosis) in rodents and ticks, only one UFI patient was seropositive (IgG) to anaplasmosis. Further characterization of *Anaplasma* species detected in the UFI patient is required to identify this pathogenic species causing human infection. Given the fact that a few *Anaplasma* species were detected from rodents and ticks in this study area such as *A. phagocytophilum*, *A. bovis*, and *A. platys*, knowing what species caused infection in humans would lead to a better understanding of the transmission dynamics among the vector, host, and reservoir enable to and to better understand its transmission in the area and reservoir host and the vector involved. Other bovine and canine ehrlichiosis were also detected in ticks. Interestingly, *Bor. miyamotoi* and *Bor. yangtzensis* were detected in rodents and *Ixodes* ticks which marks the first detection of these human pathogenic species in Thailand. More research and surveillance is needed to further characterize their prevalence and distribution in the country. *Borrelia* spp. detected in chiggers could be some other unidentified bacteria since their sequence identity to most *Borrelia* species were quite low based on *fla*B gene sequences (64.0–70.2%), while the percent identity among all reference sequences used in the alignment ranges from 69.6 to100%. While the 16S rRNA sequences of two chigger pools were 97.4% identical to some unknown *Borrelia* species and *Candidatus* Borrelia africana (Accession No. KT364339), additional analysis such as multilocus sequence typing (MLST) should be performed in order to determine whether *Borrelia* spp. detected in chiggers are new *Borrelia* species or some other bacterial genus. Since some sample types included in this study were not collected across all years of sampling (2014, 2017, and 2018) such as ectoparasites collected from domesticated mammals (2014) and UFI patients (2017), the observed pathogens reported here might not represent the true picture of pathogens shared among the vectors, reservoirs, and hosts in Bo Kluea district, Nan province. In this study, co-infection between *Anaplasma* spp. (*A. phagocytophilum, A. bovis*) and *Bartonella* spp. was observed in *Rattus* and *Bandicota* rats (2/309, 0.65%). It is unfortunate that the co-infection/co-occurrence patterns in ectoparasites could not be examined in this study due to our pooling procedure for ectoparasites which was performed immediately after they were collected from animal hosts.

Seroprevalence (IgM and IgG) for scrub typhus and rickettsiosis in UFI patients confirmed that the two diseases are highly endemic to the region, especially for scrub typhus. *O. tsutsugamushi* was present in all related samples studied and human exposure was clearly observed with high prevalence and titers (*n* = 154 with 1600, >6400 titers). Although human rickettsiosis was not detected in rodents or vectors, the levels of IgM and IgG seroprevalence for TGR and SFGR indicate the circulation of these pathogens in the area as well. Some pathogens were detected in animals and vectors but not in humans; however, seroprevalence (IgG) of the pathogens in patients indicated previous exposure in humans, such as *B. henselae*. It is worth noting that serological differentiation between *B. henselae* and *B. quintana* IgG antibody might not be possible since there could be some cross-reactivity between the two species. With *O. tsutsugamushi*, when age and sex of patients were considered, prevalence was significantly higher in 20–40 and 41–60 year-old groups, which shows the working age population having increased risk of contracting the diseases. The incidence of scrub typhus infections in humans seems to occur at higher rates during the rainy season corresponding to the time when local people start rice/corn cultivation and continues throughout the year until the harvesting season ends in October/November as the high seroprevalence in working age groups (20–60 years old) strongly supports this speculation. Moreover, the infection rate of *O. tsutsugamushi* in rodents and chiggers was highest in March just before the rainy season, followed by the increase of chigger indexes (number of chiggers per host) possibly leading to increased potential for disease transmission.

The discovery of certain bacterial pathogens was expected based on previous surveillance data and reported cases from the Ministry of Public Health. Additionally, other unexpected pathogens such as *Anaplasma* spp. and *B. quintana* (an agent causing Trench fever) were detected among UFI patients as well as *Bor. miyamotoi* in rodent populations. However, to date in-depth analyses as to how, when and where transmission occurs are lacking. Human, animal, and vector interactions play a major role in disease transmission and form a dynamic transmission cycle. Pathogens can spread from animal-to-animal or animal-to-human by several modes of transmission. Probably the most important method of transmission occurs during feeding by parasitic arthropods. This study employed NGS and metagenomics to characterize bacterial pathogens and understand their transmission in animal, human, and vector populations. Several pathogens were detected in rodent and vector populations indicating the complex ecology of bacterial pathogens and their reservoir hosts and vectors in the area close to where human activities occur which increase the risk of human–animal interface. The most apparent example is scrub typhus where *O. tsutsugamushi* was found in UFI patients, chiggers, and rodent populations. These data clearly illustrate the complex picture of pathogen transmission from animal reservoir hosts to humans via arthropod vectors. Local public health officials can effectively use the data to assist in understanding the seasonality of diseases such as scrub typhus and the populations most at risk. Information can be shared locally and preventive measures, such as repellents, can be used when appropriate. From this study, multiple bacterial pathogens known to cause human diseases in other locations were identified for the first time in Nan province. Such information is useful for local medical providers as they try to diagnose and treat patients with undifferentiated fevers. Finally, the data presented in this study effectively illustrate the utility of metagenomics in future epidemiological surveys involving multiple types of samples.

## Author Contributions

RT and AK conceived and designed the experiments. AR, NC, SP, TM, ST, and BT performed the experiments. CP, KY, and NK were the hospital staff involved in this study. RT, AK, and SD analyzed the data. RT, SD, and AK wrote the manuscript. ALR provided reagents and reviewed the manuscript.

## Disclaimer

Material has been reviewed by the Walter Reed Army Institute of Research. There is no objection to its presentation and/or publication. The opinions or assertions contained herein are the private views of the author, and are not to be construed as official, or as reflecting true views of the Department of the Army or the Department of Defense. Research was conducted under an approved animal use protocol in an AAALACi accredited facility in compliance with the Animal Welfare Act and other federal statutes and regulations relating to animals and experiments involving animals and adheres to principles stated in the “Guide for the Care and Use of Laboratory Animals”, NRC Publication, 2011 edition. The investigators have adhered to the policies for protection of human subjects as prescribed in Army Regulations 70–25.

## Conflict of Interest Statement

The authors declare that the research was conducted in the absence of any commercial or financial relationships that could be construed as a potential conflict of interest.

## Footnotes

1 http://www.clcbio.com

2 www.graphpad.com

3 https://www.qgis.org/en/site/

4 http://climate.tmd.go.th/content/category/17

5 http://www.boe.moph.go.th/boedb/surdata/index.php
