# Supplementary figures and images for: Metagenomic Approach to Characterizing Disease Epidemiology in a Disease-Endemic Environment in Northern Thailand
Source: Front Microbiol. 2019 Feb 26;10:319. doi: 10.3389/fmicb.2019.00319 (PMC6399164; doi:10.3389/fmicb.2019.00319)

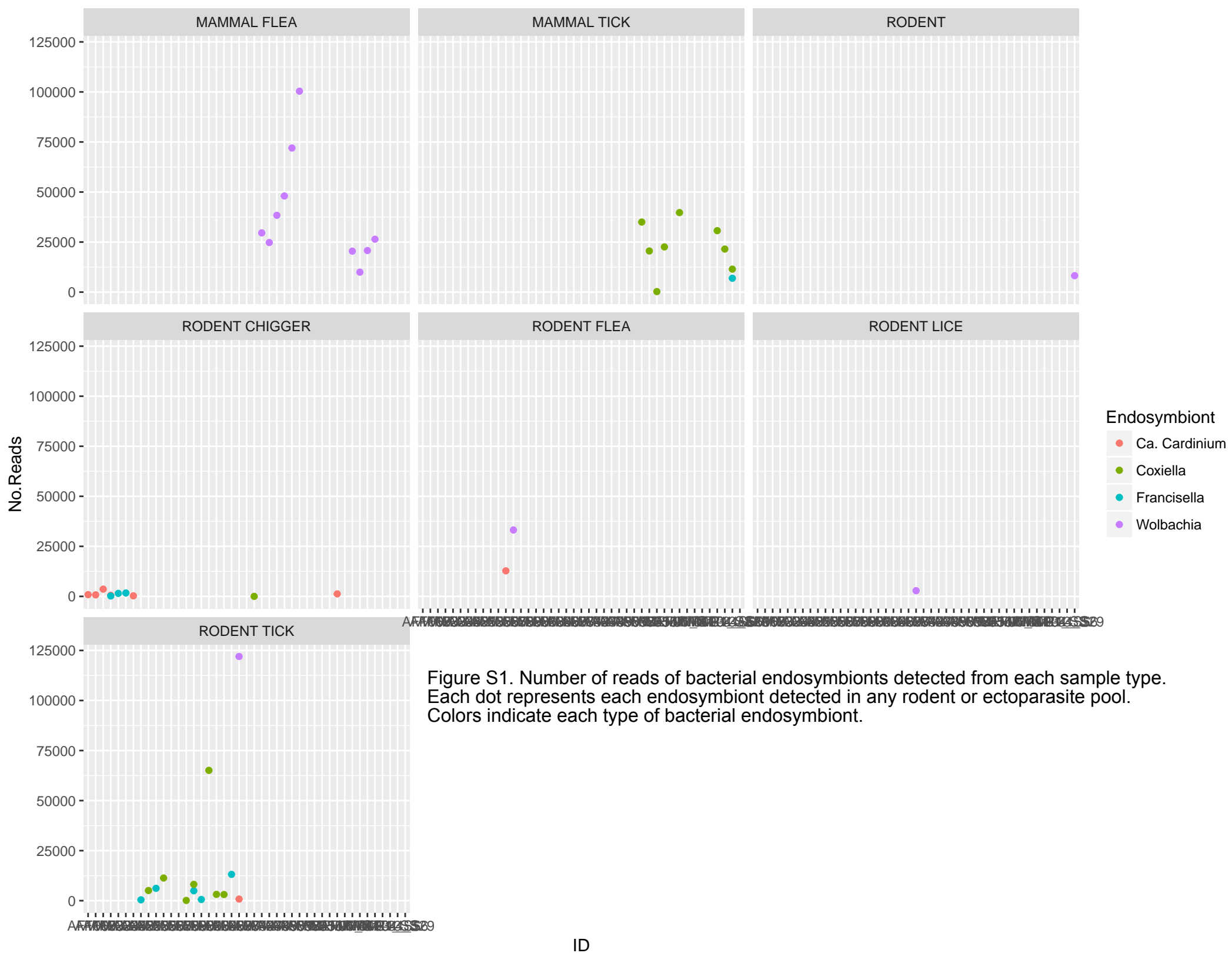

Supplement: Supplementary file 1 [file Data_Sheet_1.PDF]
