# Supplementary material for: Metagenomic Approach to Characterizing Disease Epidemiology in a Disease-Endemic Environment in Northern Thailand
Source: Front Microbiol. 2019 Feb 26;10:319. doi: 10.3389/fmicb.2019.00319 (PMC6399164; doi:10.3389/fmicb.2019.00319)

Phylogenetic analysis of pathogens detected from NGS results (red letters). All maximum likelihood trees were created using the best fit model of nucleotide substitution in the MEGA 6 program with bootstrapping (1000 replicates).

Leptospira spp. (16S)
Model: ML (K2+G)

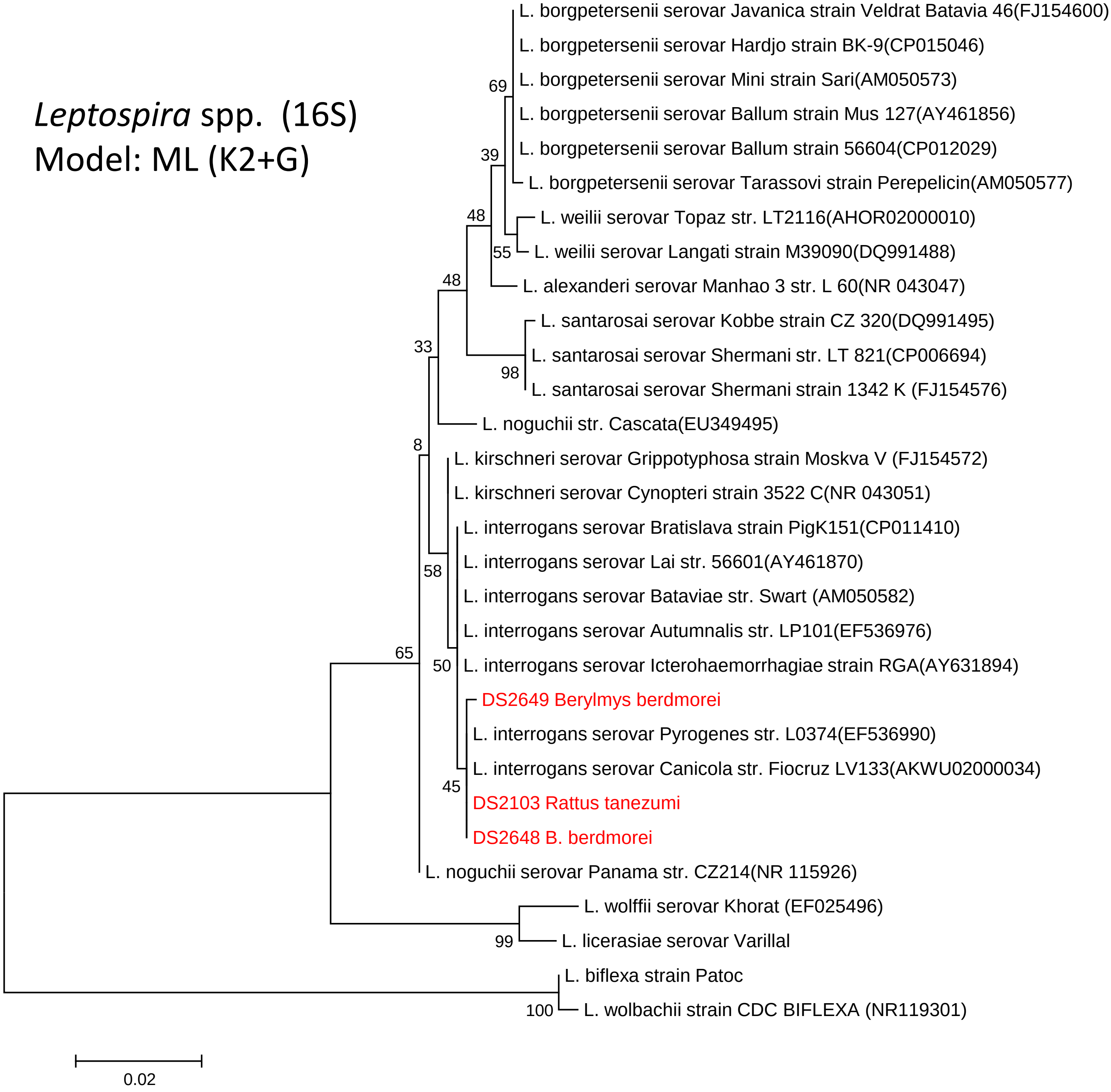

Leptospira spp. (*sec*Y)

Model: ML(HKY+G)

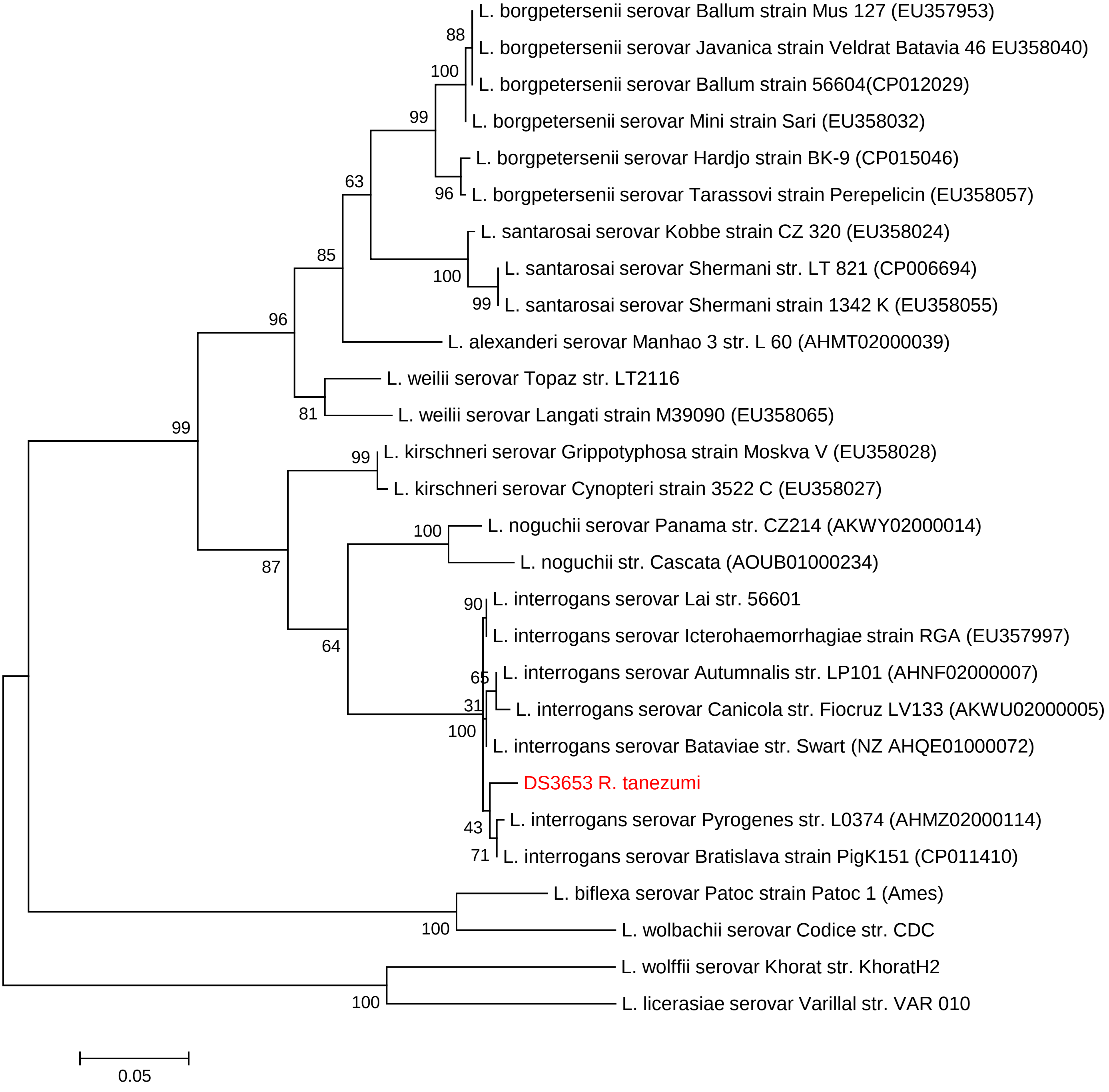

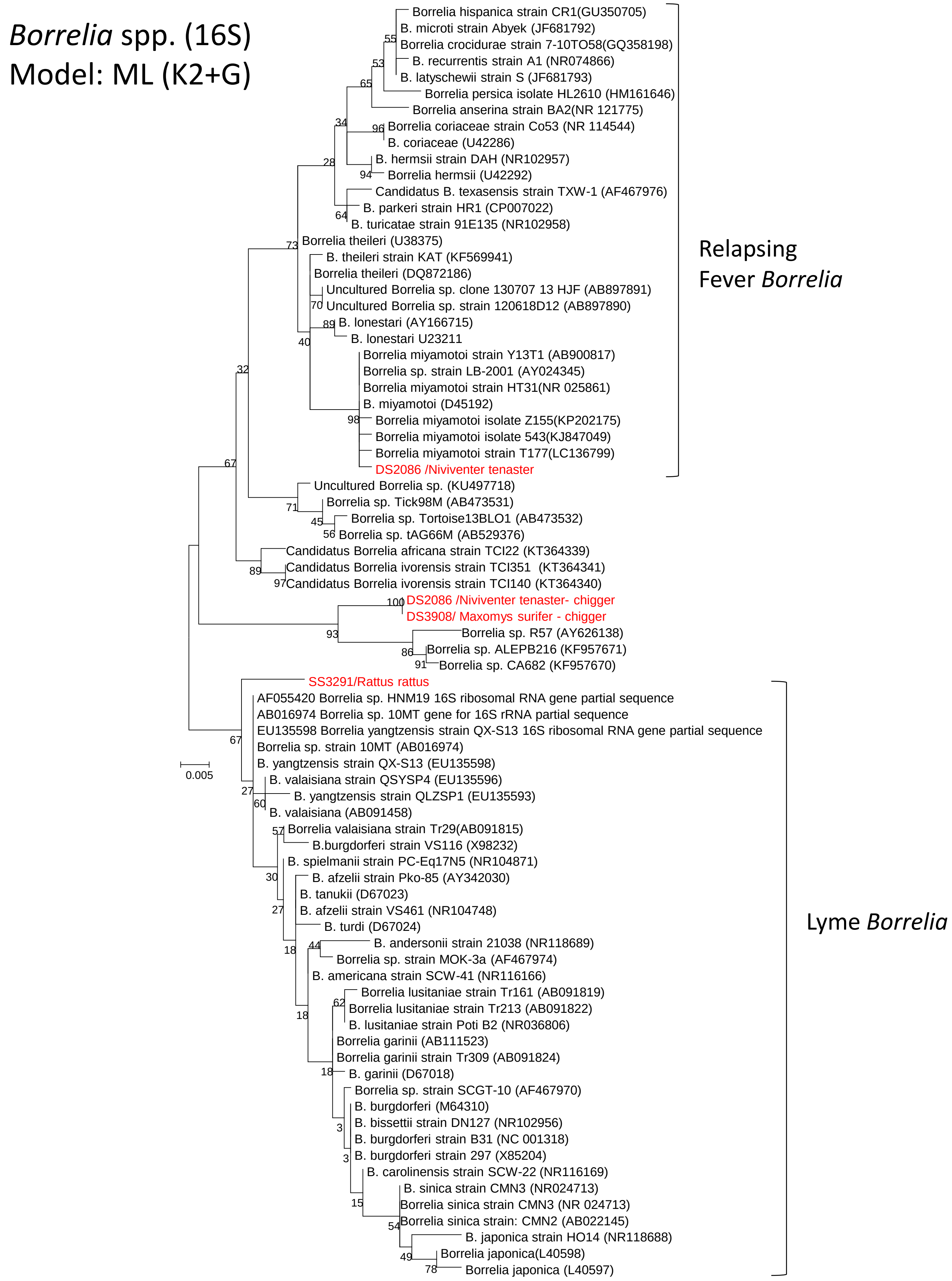
Borrelia spp. (16S)
Model: ML (K2+G)
Borrelia hispanica strain CR1(GU350705)
B. microti strain Abyek (JF681792)
Borrelia crocidurae strain 7-10TO58(GQ358198)
B. recurrentis strain A1 (NR074866)
B. latyschewii strain S (JF681793)
Borrelia persica isolate HL2610 (HM161646)
Borrelia anserina strain BA2(NR 121775)
Borrelia coriaceae strain Co53 (NR 114544)
B. coriaceae (U42286)
B. hermsii strain DAH (NR102957)
Borrelia hermsii (U42292)
Candidatus B. texasensis strain TXW-1 (AF467976)
B. parkeri strain HR1 (CP007022)
B. turicatae strain 91E135 (NR102958)
Borrelia theileri (U38375)
B. theileri strain KAT (KF569941)
Borrelia theileri (DQ872186)
Uncultured Borrelia sp. clone 130707 13 HJF (AB897891)
Uncultured Borrelia sp. strain 120618D12 (AB897890)
B. lonestari (AY166715)
B. lonestari U23211
Borrelia miyamotoi strain Y13T1 (AB900817)
Borrelia sp. strain LB-2001 (AY024345)
Borrelia miyamotoi strain HT31(NR 025861)
B. miyamotoi (D45192)
Borrelia miyamotoi isolate Z155(KP202175)
Borrelia miyamotoi isolate 543(KJ847049)
Borrelia miyamotoi strain T177(LC136799)
DS2086 /Niviventer tenaster
Relapsing Fever Borrelia
Uncultured Borrelia sp. (KU497718)
Borrelia sp. Tick98M (AB473531)
Borrelia sp. Tortoise13BLO1 (AB473532)
Borrelia sp. tAG66M (AB529376)
Candidatus Borrelia africana strain TCI22 (KT364339)
Candidatus Borrelia ivorensis strain TCI351 (KT364341)
Candidatus Borrelia ivorensis strain TCI140 (KT364340)
DS2086 /Niviventer tenaster- chigger
DS3908/ Maxomys surifer - chigger
Borrelia sp. R57 (AY626138)
Borrelia sp. ALEPB216 (KF957671)
Borrelia sp. CA682 (KF957670)
SS3291/Rattus rattus
AF055420 Borrelia sp. HNM19 16S ribosomal RNA gene partial sequence
AB016974 Borrelia sp. 10MT gene for 16S rRNA partial sequence
EU135598 Borrelia yangtzensis strain QX-S13 16S ribosomal RNA gene partial sequence
Borrelia sp. strain 10MT (AB016974)
B. yangtzensis strain QX-S13 (EU135598)
B. valaisiana strain QSYSP4 (EU135596)
B. yangtzensis strain QLZSP1 (EU135593)
B. valaisiana (AB091458)
Borrelia valaisiana strain Tr29(AB091815)
B.burgdorferi strain VS116 (X98232)
B. spielmanii strain PC-Eq17N5 (NR104871)
B. afzelii strain Pko-85 (AY342030)
B. tanukii (D67023)
B. afzelii strain VS461 (NR104748)
B. turdi (D67024)
B. andersonii strain 21038 (NR118689)
Borrelia sp. strain MOK-3a (AF467974)
B. americana strain SCW-41 (NR116166)
Borrelia lusitaniae strain Tr161 (AB091819)
Borrelia lusitaniae strain Tr213 (AB091822)
B. lusitaniae strain Poti B2 (NR036806)
Borrelia garinii (AB111523)
Borrelia garinii strain Tr309 (AB091824)
B. garinii (D67018)
Borrelia sp. strain SCGT-10 (AF467970)
B. burgdorferi (M64310)
B. bissettii strain DN127 (NR102956)
B. burgdorferi strain B31 (NC 001318)
B. burgdorferi strain 297 (X85204)
B. carolinensis strain SCW-22 (NR116169)
B. sinica strain CMN3 (NR024713)
Borrelia sinica strain CMN3 (NR 024713)
Borrelia sinica strain: CMN2 (AB022145)
B. japonica strain HO14 (NR118688)
Borrelia japonica(L40598)
Borrelia japonica (L40597)
Lyme Borrelia
0.005

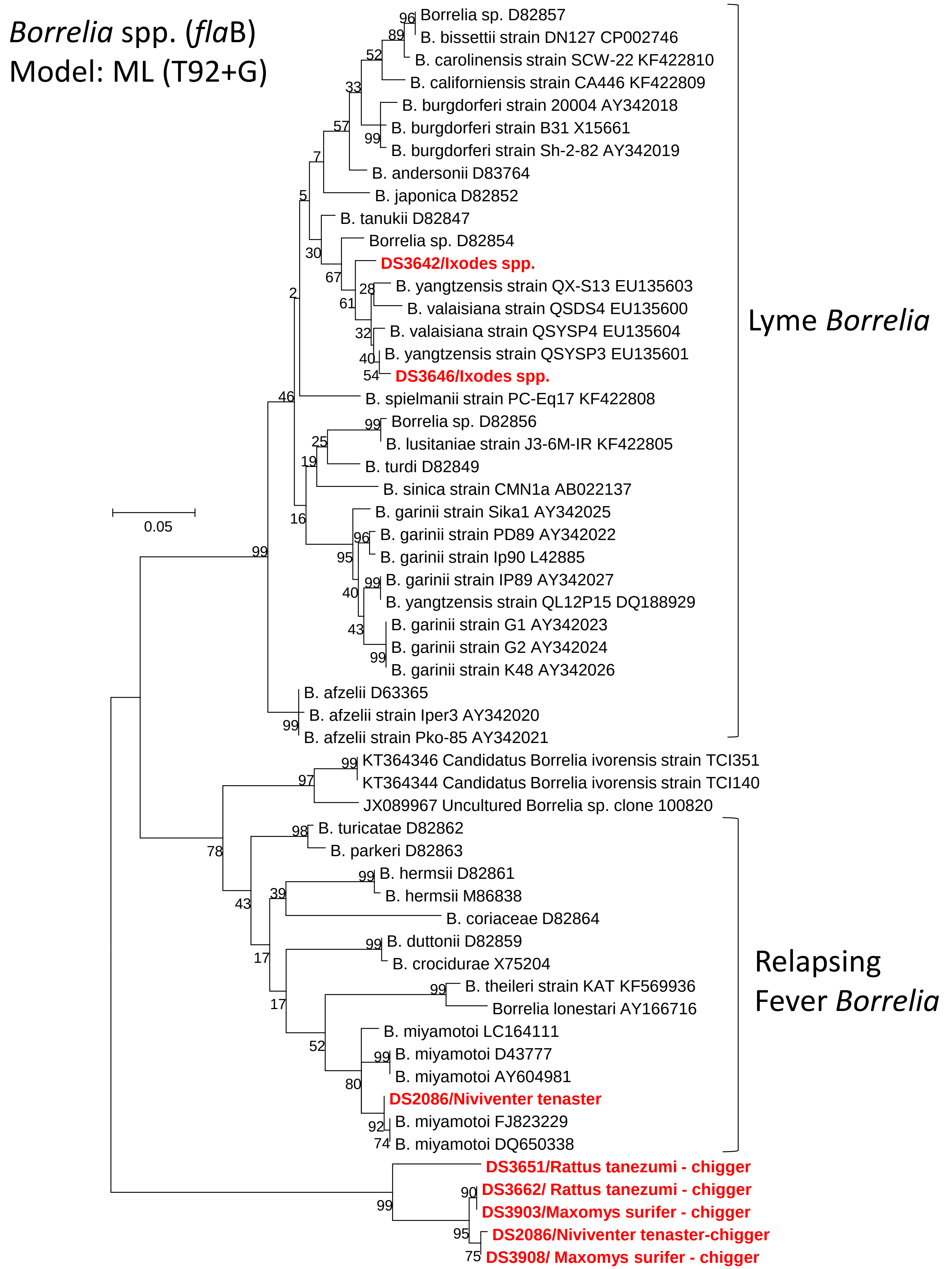
Borrelia spp. (flaB)
Model: ML (T92+G)
Borrelia sp. D82857
B. bissettii strain DN127 CP002746
B. carolinensis strain SCW-22 KF422810
B. californiensis strain CA446 KF422809
B. burgdorferi strain 20004 AY342018
B. burgdorferi strain B31 X15661
B. burgdorferi strain Sh-2-82 AY342019
B. andersonii D83764
B. japonica D82852
B. tanukii D82847
Borrelia sp. D82854
DS3642/Ixodes spp.
B. yangtzensis strain QX-S13 EU135603
B. valaisiana strain QSDS4 EU135600
B. valaisiana strain QSYSP4 EU135604
B. yangtzensis strain QSYSP3 EU135601
DS3646/Ixodes spp.
B. spielmanii strain PC-Eq17 KF422808
Borrelia sp. D82856
B. lusitaniae strain J3-6M-IR KF422805
B. turdi D82849
B. sinica strain CMN1a AB022137
B. garinii strain Sika1 AY342025
B. garinii strain PD89 AY342022
B. garinii strain Ip90 L42885
B. garinii strain IP89 AY342027
B. yangtzensis strain QL12P15 DQ188929
B. garinii strain G1 AY342023
B. garinii strain G2 AY342024
B. garinii strain K48 AY342026
B. afzelii D63365
B. afzelii strain Iper3 AY342020
B. afzelii strain Pko-85 AY342021
KT364346 Candidatus Borrelia ivorensis strain TCI351
KT364344 Candidatus Borrelia ivorensis strain TCI140
JX089967 Uncultured Borrelia sp. clone 100820
B. turicatae D82862
B. parkeri D82863
B. hermsii D82861
B. hermsii M86838
B. coriaceae D82864
B. duttonii D82859
B. crocidurae X75204
B. theileri strain KAT KF569936
Borrelia lonestari AY166716
B. miyamotoi LC164111
B. miyamotoi D43777
B. miyamotoi AY604981
DS2086/Niviventer tenaster
B. miyamotoi FJ823229
B. miyamotoi DQ650338
DS3651/Rattus tanezumi - chigger
DS3662/ Rattus tanezumi - chigger
DS3903/Maxomys surifer - chigger
DS2086/Niviventer tenaster-chigger
DS3908/ Maxomys surifer - chigger
Lyme Borrelia
Relapsing Fever Borrelia
0.05
96
89
52
33
57
99
7
5
30
67
28
61
32
40
54
2
46
99
25
19
16
96
95
99
40
43
99
99
99
97
98
78
99
39
43
99
17
99
17
52
99
80
92
74
90
99
95
75

Coxiella spp. (16S)
Model: ML(K2+G)

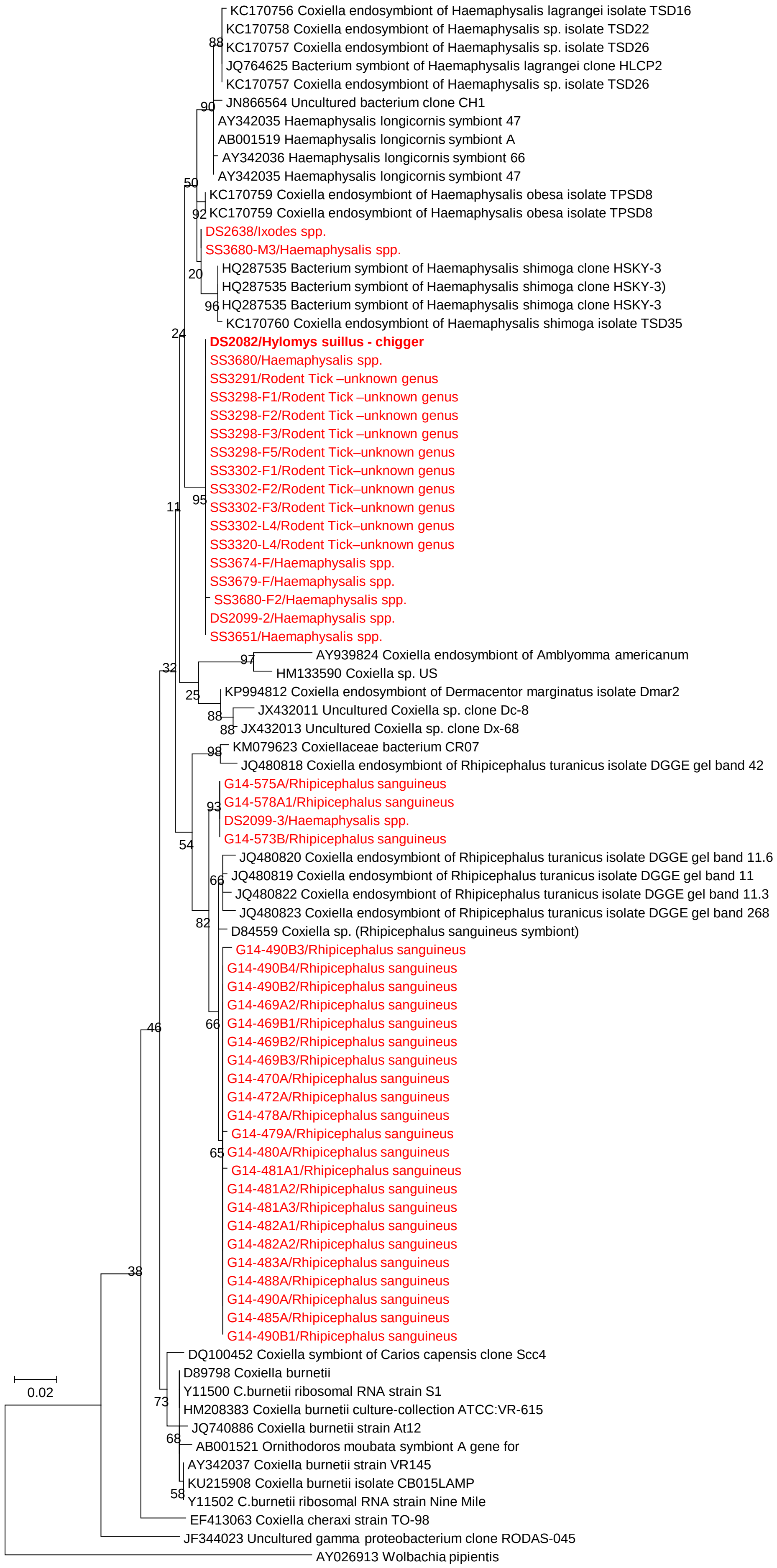

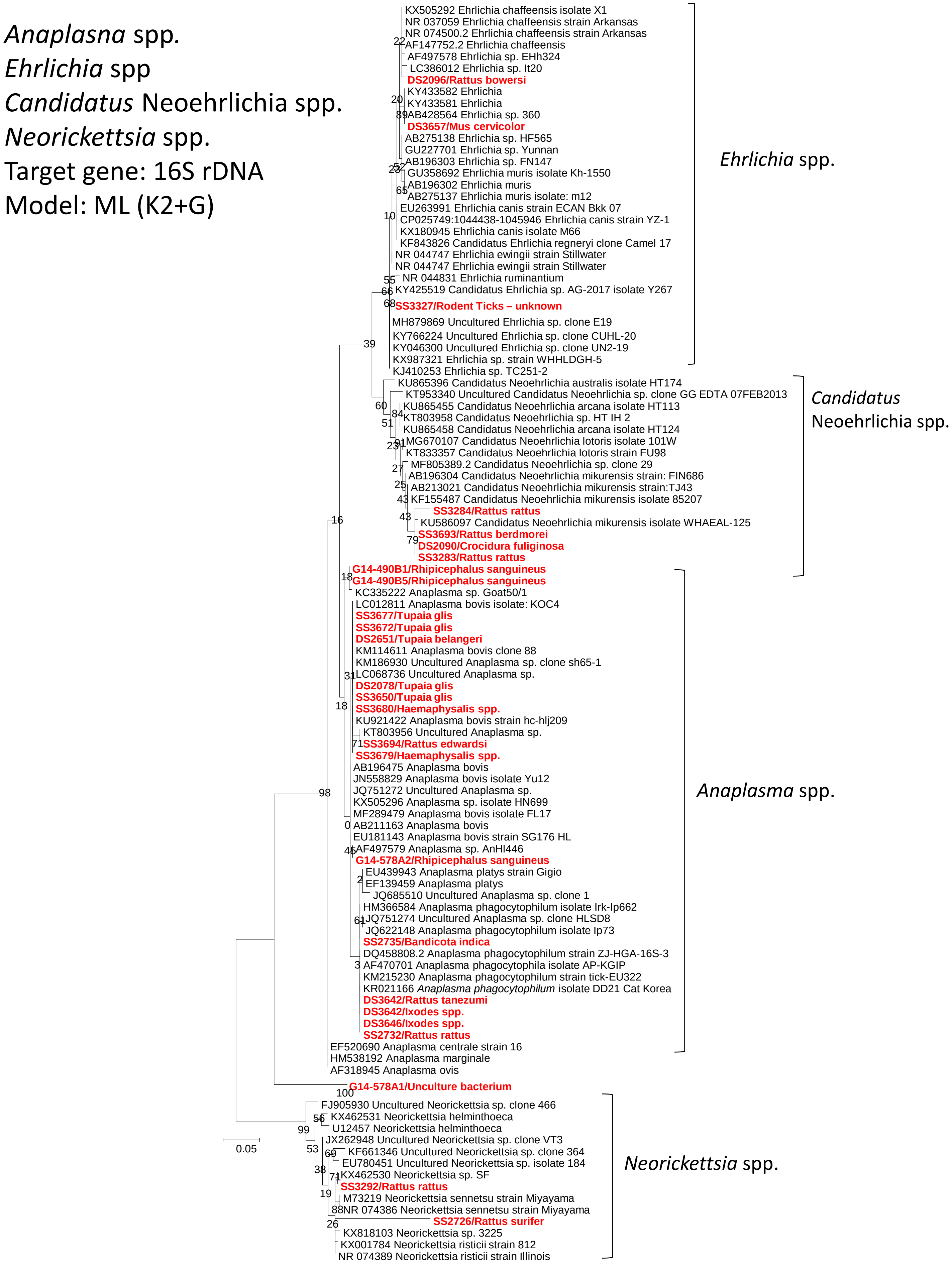
Anaplasna spp.
Ehrlichia spp
Candidatus Neoehrlichia spp.
Neorickettsia spp.
Target gene: 16S rDNA
Model: ML (K2+G)
KX505292 Ehrlichia chaffeensis isolate X1
NR 037059 Ehrlichia chaffeensis strain Arkansas
NR 074500.2 Ehrlichia chaffeensis strain Arkansas
AF147752.2 Ehrlichia chaffeensis
AF497578 Ehrlichia sp. EHh324
LC386012 Ehrlichia sp. It20
DS2096/Rattus bowersi
KY433582 Ehrlichia
KY433581 Ehrlichia
AB428564 Ehrlichia sp. 360
DS3657/Mus cervicolor
AB275138 Ehrlichia sp. HF565
GU227701 Ehrlichia sp. Yunnan
AB196303 Ehrlichia sp. FN147
GU358692 Ehrlichia muris isolate Kh-1550
AB196302 Ehrlichia muris
AB275137 Ehrlichia muris isolate: m12
EU263991 Ehrlichia canis strain ECAN Bkk 07
CP025749:1044438-1045946 Ehrlichia canis strain YZ-1
KX180945 Ehrlichia canis isolate M66
KF843826 Candidatus Ehrlichia regneryi clone Camel 17
NR 044747 Ehrlichia ewingii strain Stillwater
NR 044747 Ehrlichia ewingii strain Stillwater
NR 044831 Ehrlichia ruminantium
KY425519 Candidatus Ehrlichia sp. AG-2017 isolate Y267
SS3327/Rodent Ticks – unknown
MH879869 Uncultured Ehrlichia sp. clone E19
KY766224 Uncultured Ehrlichia sp. clone CUHL-20
KY046300 Uncultured Ehrlichia sp. clone UN2-19
KX987321 Ehrlichia sp. strain WHHLDGH-5
KJ410253 Ehrlichia sp. TC251-2
Ehrlichia spp.
KU865396 Candidatus Neoehrlichia australis isolate HT174
KT953340 Uncultured Candidatus Neoehrlichia sp. clone GG EDTA 07FEB2013
KU865455 Candidatus Neoehrlichia arcana isolate HT113
KT803958 Candidatus Neoehrlichia sp. HT IH 2
KU865458 Candidatus Neoehrlichia arcana isolate HT124
MG670107 Candidatus Neoehrlichia lotoris isolate 101W
KT833357 Candidatus Neoehrlichia lotoris strain FU98
MF805389.2 Candidatus Neoehrlichia sp. clone 29
AB196304 Candidatus Neoehrlichia mikurensis strain: FIN686
AB213021 Candidatus Neoehrlichia mikurensis strain:TJ43
KF155487 Candidatus Neoehrlichia mikurensis isolate 85207
SS3284/Rattus rattus
KU586097 Candidatus Neoehrlichia mikurensis isolate WHAEAL-125
SS3693/Rattus berdmorei
DS2090/Crocidura fuliginosa
SS3283/Rattus rattus
Candidatus
Neoehrlichia spp.
G14-490B1/Rhipicephalus sanguineus
G14-490B5/Rhipicephalus sanguineus
KC335222 Anaplasma sp. Goat50/1
LC012811 Anaplasma bovis isolate: KOC4
SS3677/Tupaia glis
SS3672/Tupaia glis
DS2651/Tupaia belangeri
KM114611 Anaplasma bovis clone 88
KM186930 Uncultured Anaplasma sp. clone sh65-1
LC068736 Uncultured Anaplasma sp.
DS2078/Tupaia glis
SS3650/Tupaia glis
SS3680/Haemaphysalis spp.
KU921422 Anaplasma bovis strain hc-hlj209
KT803956 Uncultured Anaplasma sp.
SS3694/Rattus edwardsi
SS3679/Haemaphysalis spp.
AB196475 Anaplasma bovis
JN558829 Anaplasma bovis isolate Yu12
JQ751272 Uncultured Anaplasma sp.
KX505296 Anaplasma sp. isolate HN699
MF289479 Anaplasma bovis isolate FL17
AB211163 Anaplasma bovis
EU181143 Anaplasma bovis strain SG176 HL
AF497579 Anaplasma sp. AnHl446
G14-578A2/Rhipicephalus sanguineus
EU439943 Anaplasma platys strain Gigio
EF139459 Anaplasma platys
JQ685510 Uncultured Anaplasma sp. clone 1
HM366584 Anaplasma phagocytophilum isolate Irk-Ip662
JQ751274 Uncultured Anaplasma sp. clone HLSD8
JQ622148 Anaplasma phagocytophilum isolate Ip73
SS2735/Bandicota indica
DQ458808.2 Anaplasma phagocytophilum strain ZJ-HGA-16S-3
AF470701 Anaplasma phagocytophila isolate AP-KGIP
KM215230 Anaplasma phagocytophilum strain tick-EU322
KR021166 Anaplasma phagocytophilum isolate DD21 Cat Korea
DS3642/Rattus tanezumi
DS3642/Ixodes spp.
DS3646/Ixodes spp.
SS2732/Rattus rattus
EF520690 Anaplasma centrale strain 16
HM538192 Anaplasma marginale
AF318945 Anaplasma ovis
Anaplasma spp.
G14-578A1/Unculture bacterium
FJ905930 Uncultured Neorickettsia sp. clone 466
KX462531 Neorickettsia helminthoeca
U12457 Neorickettsia helminthoeca
JX262948 Uncultured Neorickettsia sp. clone VT3
KF661346 Uncultured Neorickettsia sp. clone 364
EU780451 Uncultured Neorickettsia sp. isolate 184
KX462530 Neorickettsia sp. SF
SS3292/Rattus rattus
M73219 Neorickettsia sennetsu strain Miyayama
NR 074386 Neorickettsia sennetsu strain Miyayama
SS2726/Rattus surifer
KX818103 Neorickettsia sp. 3225
KX001784 Neorickettsia risticii strain 812
NR 074389 Neorickettsia risticii strain Illinois
Neorickettsia spp.
0.05

Anaplasma & *Ehrlichia* spp. (*gro*EL)
Model:T92+G

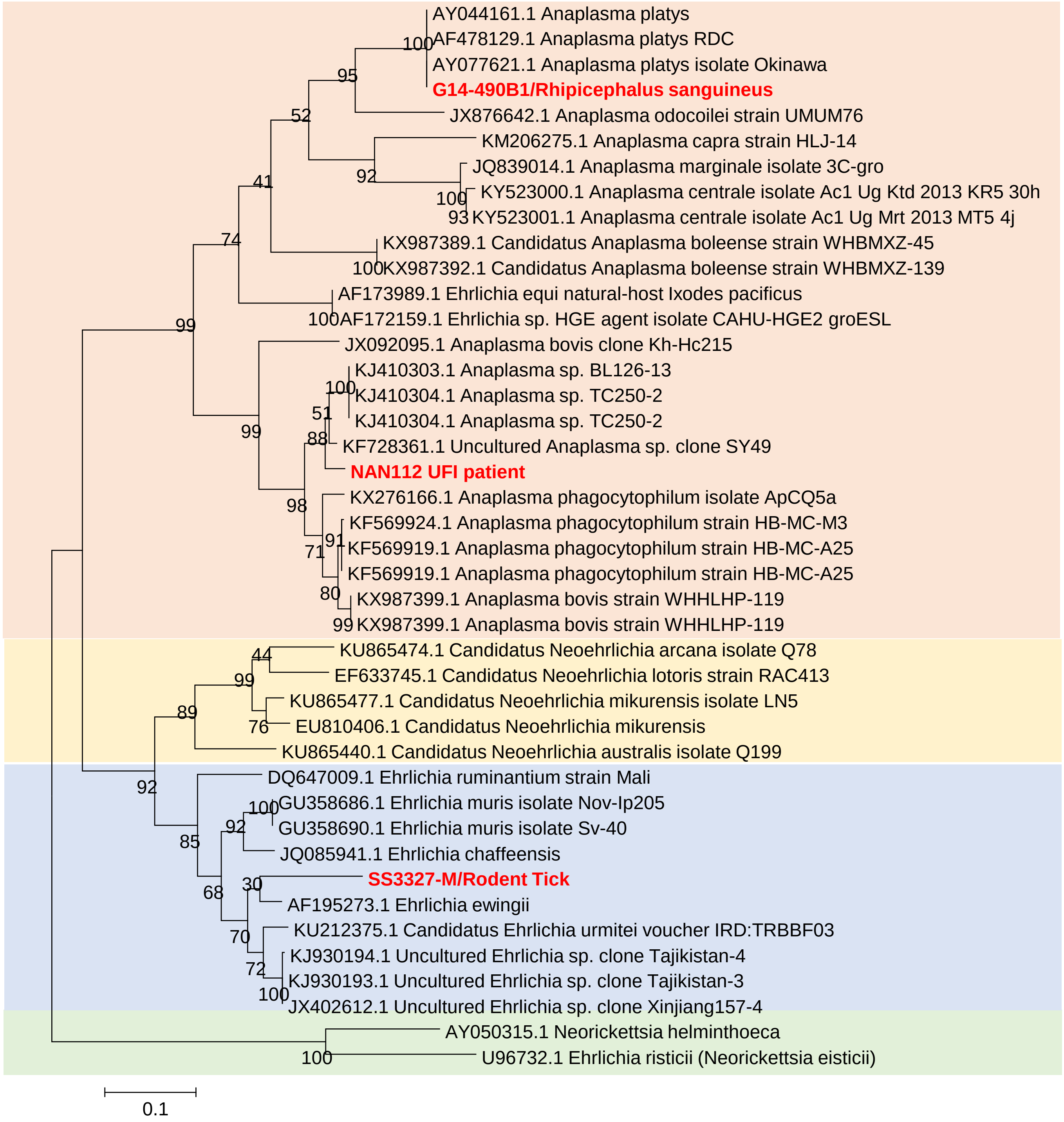

Bartonella spp. (*ssr*A)

Model: ML (K2+G)

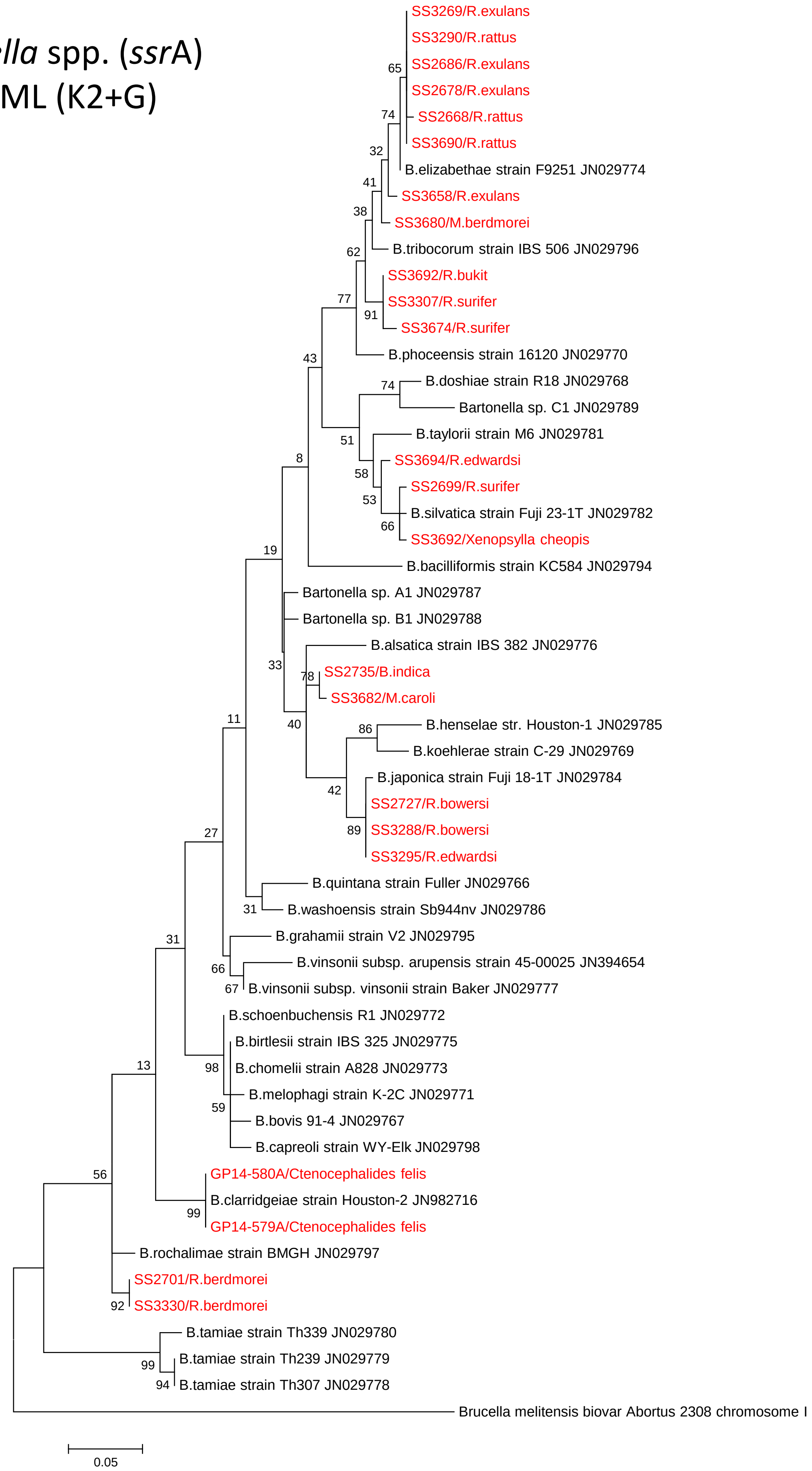

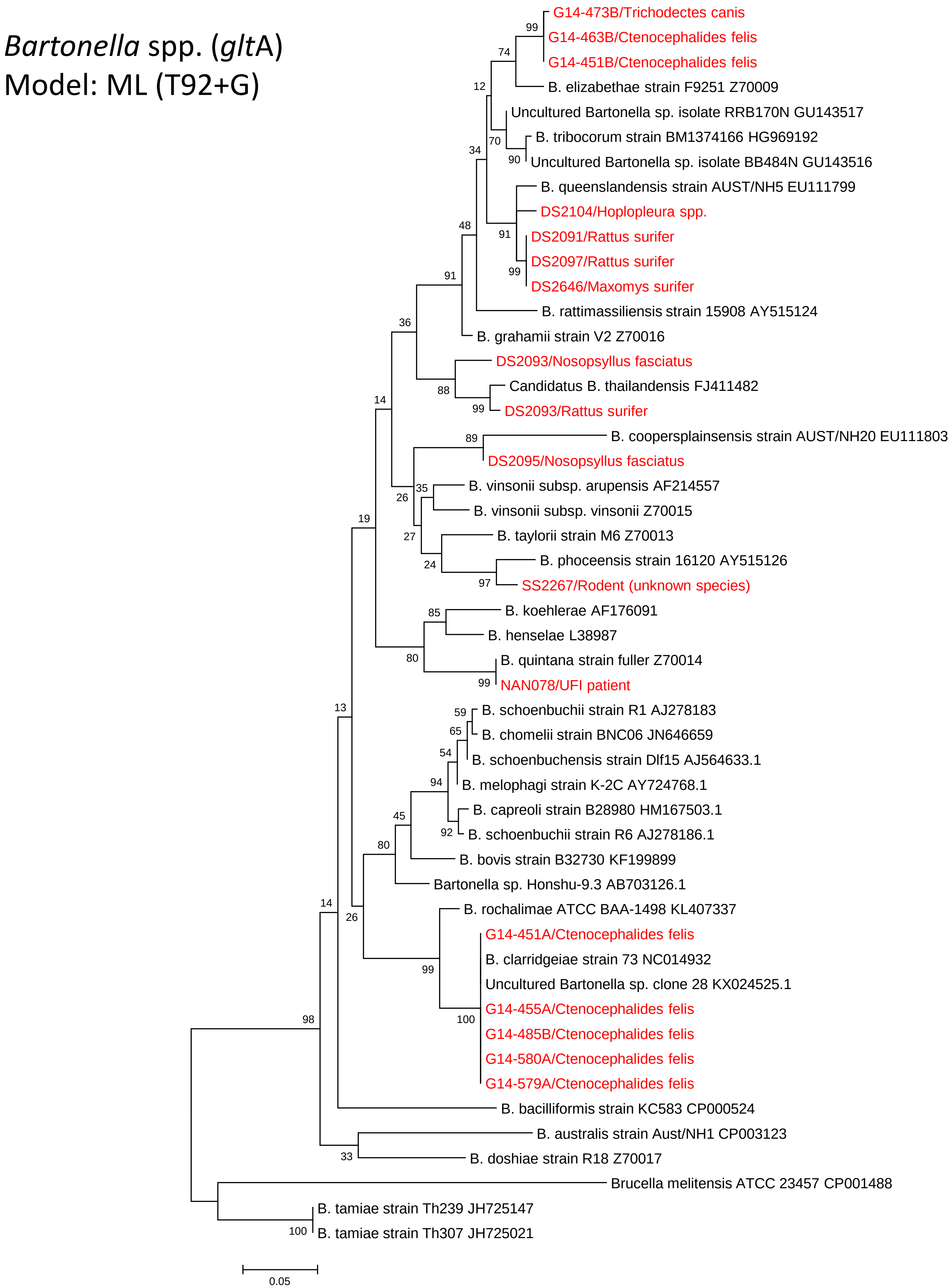
Bartonella spp. (*glt*A)
Model: ML (T92+G)
G14-473B/Trichodectes canis
G14-463B/Ctenocephalides felis
G14-451B/Ctenocephalides felis
B. elizabethae strain F9251 Z70009
Uncultured Bartonella sp. isolate RRB170N GU143517
B. tribocorum strain BM1374166 HG969192
Uncultured Bartonella sp. isolate BB484N GU143516
B. queenslandensis strain AUST/NH5 EU111799
DS2104/Hoplopleura spp.
DS2091/Rattus surifer
DS2097/Rattus surifer
DS2646/Maxomys surifer
B. rattimassiliensis strain 15908 AY515124
B. grahamii strain V2 Z70016
DS2093/Nosopsyllus fasciatus
Candidatus B. thailandensis FJ411482
DS2093/Rattus surifer
B. coopersplainsensis strain AUST/NH20 EU111803
DS2095/Nosopsyllus fasciatus
B. vinsonii subsp. arupensis AF214557
B. vinsonii subsp. vinsonii Z70015
B. taylorii strain M6 Z70013
B. phoceensis strain 16120 AY515126
SS2267/Rodent (unknown species)
B. koehlerae AF176091
B. henselae L38987
B. quintana strain fuller Z70014
NAN078/UFI patient
B. schoenbuchii strain R1 AJ278183
B. chomelii strain BNC06 JN646659
B. schoenbuchensis strain Dlf15 AJ564633.1
B. melophagi strain K-2C AY724768.1
B. capreoli strain B28980 HM167503.1
B. schoenbuchii strain R6 AJ278186.1
B. bovis strain B32730 KF199899
Bartonella sp. Honshu-9.3 AB703126.1
B. rochalimae ATCC BAA-1498 KL407337
G14-451A/Ctenocephalides felis
B. clarridgeiae strain 73 NC014932
Uncultured Bartonella sp. clone 28 KX024525.1
G14-455A/Ctenocephalides felis
G14-485B/Ctenocephalides felis
G14-580A/Ctenocephalides felis
G14-579A/Ctenocephalides felis
B. bacilliformis strain KC583 CP000524
B. australis strain Aust/NH1 CP003123
B. doshiae strain R18 Z70017
Brucella melitensis ATCC 23457 CP001488
B. tamiae strain Th239 JH725147
B. tamiae strain Th307 JH725021
99
74
12
70
90
34
91
99
48
91
36
88
99
14
89
35
26
27
24
97
19
85
80
99
59
65
54
94
92
45
80
13
26
99
100
14
98
33
100
0.05

Bartonella spp. (*nuo*G)
Model: ML (T92+G)

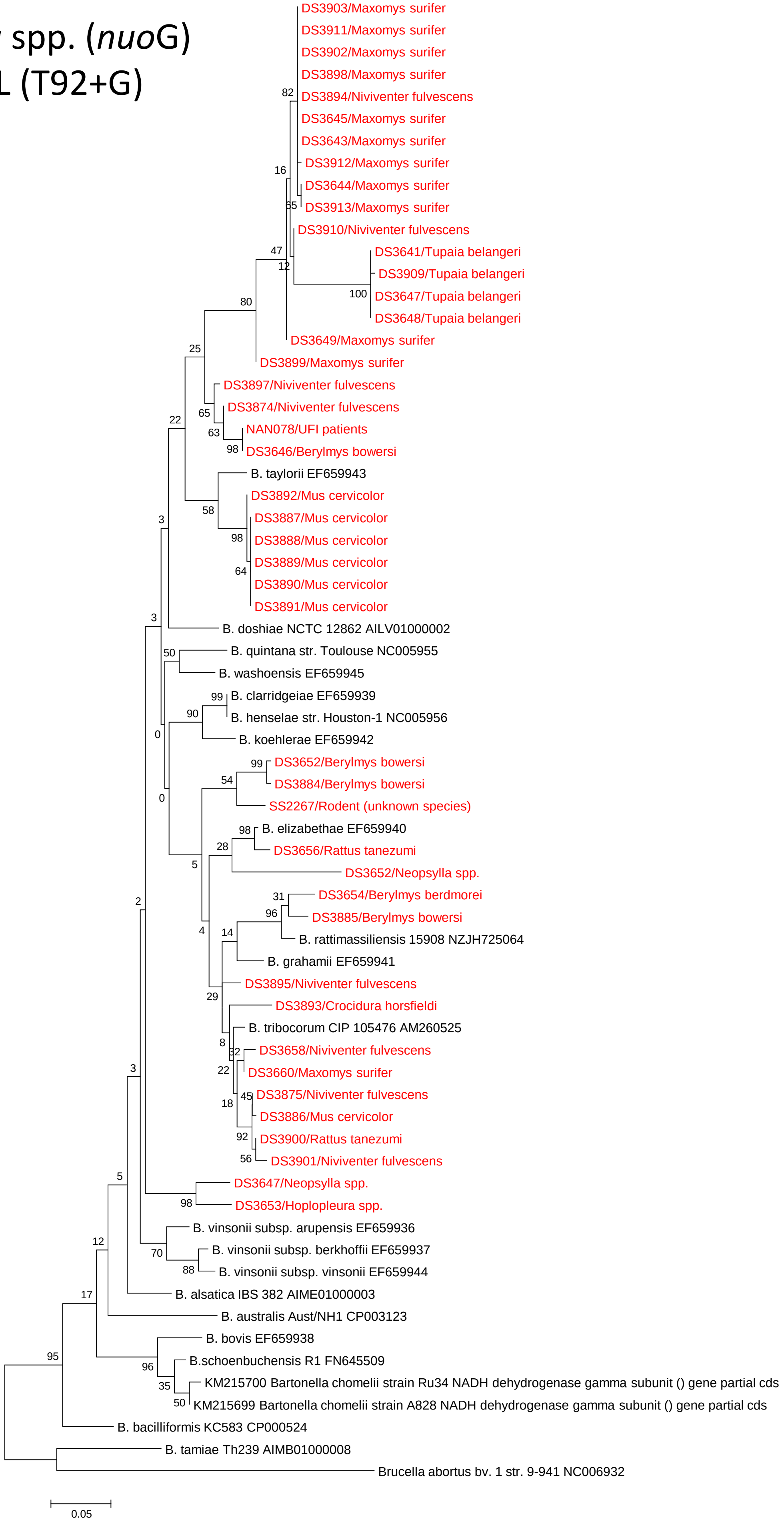

Rickettsia spp. (*glt*A)
Model: ML(T92+G)

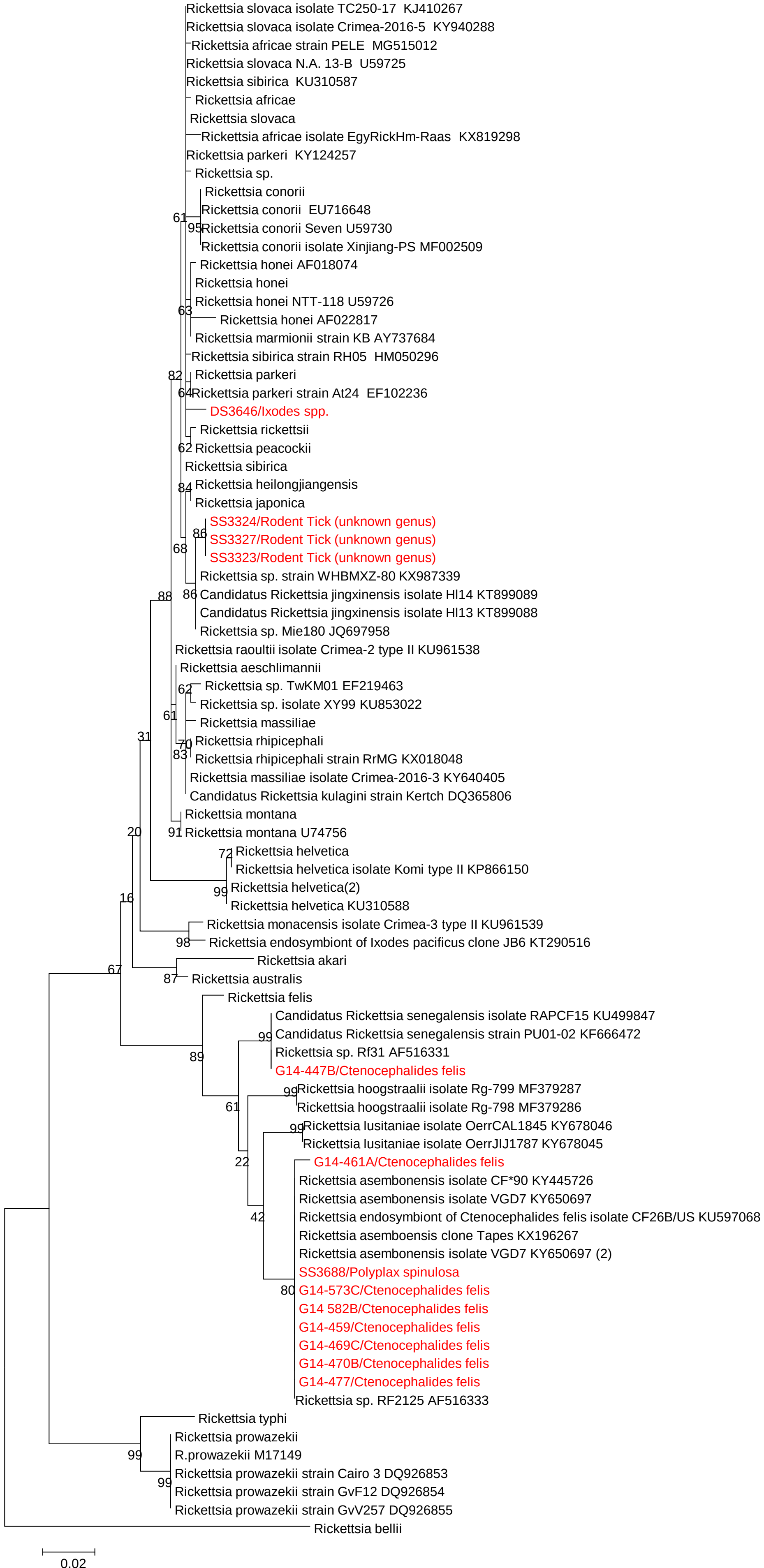

Supplement: Supplementary file 2 [file Data_Sheet_2.pdf]
